# Panel and geospatial data for U.S. FDIC insured banks fiduciary activities and annual performance analyses over the periods 2016 to 2018

**DOI:** 10.1016/j.dib.2019.104358

**Published:** 2019-08-06

**Authors:** Ibrahim Niankara

**Affiliations:** College of Business, Al Ain University of Science and Technology, Abu Dhabi, United Arab Emirates

**Keywords:** Banking regulations, Dodd-frank act, Governmental oversight, FDIC insurance, Fiduciary activities, Financial reporting

## Abstract

Created in 1934, the Federal Deposit Insurance Corporation (FDIC) is the independent agency of the United States Government tasked to protect depositors of insured banks located in the U.S. against the loss of their deposits in case of bank failure. To achieve this objective, the FDIC collects data on banks condition and income through quarterly Financial Report calls, which are then publicly released as “Statistics on Depository Institutions (SDI)”. The present data article, as a follow up to Niankara and Ismail, 2019 that focuses on U.S. banks’ exposure to foreign counterparty risk, describes an extract from the quarterly SDIs that is compiled into a panel of 16209 observations on 5403 U.S. FDIC insured Banks, observed over the three-year periods of 2016, 2017 and 2018. Since our objective is to bring forth a useful data source for analyzing U.S. FDIC banks fiduciary activities and annual performance changes over the past recent years, our constructed sample contains all FDIC insured banks with end of year (4th quarter) financial reporting for each of the 3 fiscal years 2016-2018. We further supplement this data with U.S. county and state level geospatial data that allow analysts and business researchers to address questions with temporal significance, but also spatial relevance through appropriate modelling and mapping of the data. Finally, we demonstrate the usability of the data using R based descriptive analytics, with computer codes provided for prospective Analysts and business researchers.

**Table:** Specifications Table

Subject area	*Economics*
More specific subject area	*Business Economics, Financial Economics, Applied Econometrics*
Type of data	*Tables*
How data was acquired	*from the quarterly reports of the “Statistics on Depository Institutions (SDI)*
Data format	*Raw Excel, and R formatted data sets*
Experimental factors	*Banks' Demographic Information, Assets and Liabilities, Performance and Condition Ratios, and Total: Debt Securities, Deposits, Interest Expense, Interest Income, Managed Assets held in Fiduciary Accounts, Unused Commitments, Transaction Accounts, and US government obligations.*
Experimental features	*Panel (Longitudinal) data of 16209 observations on 5403 FDIC Banks with fourth quarter financial reporting over the 3 consecutive years of 2016, 2017, and 2018. Along with U.S. County and State level geospatial data.*
Data source location	*Data collection covers “all states” of the United States. With the FDIC Act defining “state” as any State of the United States, the District of Columbia, and any territory of the United States, Puerto Rico, Guam, American Samoa, the Trust Territory of the Pacific Islands, the Virgin Island, and the Northern Mariana Islands.*
Data accessibility	*The Data is with the article, and also accessible through Mendeley data:* *Niankara, Ibrahim (2019), “A Panel Data for U.S. FDIC insured Banks Annual Performance Analysis over the periods 2016 to 2018. ”, Mendeley Data, v1*https://doi.org/10.17632/jbvd7cvw96.1[1]

**Table:** 

**Value of the data** • This data provides Analysts and Business researchers with a unique opportunity to investigate patterns, and trends in United States FDIC insured financial institutions' performances using descriptive analytics. • The data is useful for research in areas including Banks performance, efficiency, and risk management; • It also allows investigations of banks' fiduciary activities at the extensive and intensive margins, and their relationship with banks' performance, efficiency and risk management strategies. • Researchers could use the data to address questions of temporal significance, but also of spatial relevance to the US banking industry. • Finally, the data could also inspire other works, which might consider expending the data coverage to periods before 2016, while adding state and county level macro-economic data, for much richer analyses that put the banking sector within the overall context of the US economy.

## 1 Data

The Federal Deposit Insurance Corporation (FDIC) is the independent agency of the United States Government tasked to protect depositors of insured banks located in the United States against the loss of their deposits in case of insured bank's failure [2]. To facilitate its oversight and regulatory missions, the FDIC collects Quarterly data on banks condition and income through its Thrift Financial Report (TFR) calls, which are filed with the Office of the comptroller of the Currency (OCC), as of the close of business on the last calendar quarter, in accordance with the Dodd-Frank Act [2]. The collected data is subsequently released to the general public by the FDIC. The current data availability at the FDIC's website covers from as early as 4th quarter 1992 to 4th quarter 2018 [3]. Each quarterly report is provided as a Zipped file containing 61 comma separated variables (.csv) data Files, describing the various aspect of FDIC Bank performances. Combined together, these 61 “csv” data files provide the full quarterly data of all reporting FDIC insured banks during that quarter.

Given our interest in extending the analysis in [7], which focused exclusively on U.S. global banks' performances outside of the country, we now turn our attention to U.S. banks' fiduciary activities and annual performances overtime inside the country, by focusing our data query on the 4th Quarter reports for the most recent three years of reporting, namely (Q4-2016, Q4-2017, and Q4-2018). After downloading these three zipped files of 61 “csv” data files each, we extracted from each unzipped folder, the 10 “csv” data files describing the aspect of FDIC banks fiduciary activities and performances of interest to us. These include:

• All_Reports_XXXX1231_Assets and Liabilities
• All_Reports_ XXXX1231_Performance and Condition Ratios
• All_Reports_ XXXX1231_Total Debt Securities
• All_Reports_ XXXX1231_Total Deposits
• All_Reports_ XXXX1231_Total Interest Expense
• All_Reports_ XXXX1231_Total Interest Income
• All_Reports_ XXXX1231_Total Managed Assets held in Fiduciary Accounts
• All_Reports_ XXXX1231_Total Unused Commitments
• All_Reports_ XXXX1231_Transaction Accounts
• All_Reports_ XXXX1231_U.S. Government Obligations

Where the “XXXX” represents the year index of the file (2016, 2017, 2018). From each of the 10 files we selected our variables of interest, and merged these variables into a single cross-sectional report file under the file name “Z_combXXXX” for each of the three years. In this way we had “Z_Comb2016”, “Z_Comb2017”, and “Z_Comb2018” representing the end of year reports of all FDIC insured banks during the fiscal years 2016, 2017 and 2018 respectively. Using Excel, these three cross-sectional data sets were then combined into a single Panel data in long form, named “Z_CombPanel” and spanning the three fiscal years 2016–2018. This excel file was then imported into the R statistical Software, and saved into an R object Named “CombFDICBanPanel” [1].

This version of the data is contained in “folder 0” of the attached supplementary materials. After further treatments of the data as described in the “material and methods” section below, the resulting final data “CombFDICBankPanelC” is retrievable from “folder 1” of the attached supplementary materials. “Folder 2” of the supplementary materials contains the state level aggregated data without (statesOutcomeDat) and with (statesOutcomeDat1) geospatial information, which are used for the mappings in Fig. 3, Fig. 4, Fig. 5, Fig. 6, Fig. 7. Although not explicitly described in the article, the county level aggregated data with (countiesOutcomeDat2) and without (countiesOutcomeDat) geospatial information are retrievable from “folder 3”. “Folder 4” on the other hand, contains the original U.S. state level “gadm36_USA_1_sf.rds” and county level “gadm36_USA_2_sf.rds” spatial meta data downloaded from the GADM library for merging with the treated bank performances data. Finally the last folder “Folder 5” in the attached supplementary materials, contains the aggregated data summaries across respectively bank charter class “bkclassOutcomeDat”, FDIC supervisory regions “fdicdbsOutcomeDat”, asset concentration hierarchy “specgrpOutcomeDat”, and data collection period/year “yearOutcomeDat”.

## 2 Experimental design, materials, and methods

The subset of data (variables) described in the present article is extracted from the R data object “CombFDICBankPanel” in [1]. Using this initial data file, and the R statistical Software [4], we proceeded to further treatments of the data into its final version “CombFDICBankPanelC” presented in more details here. The applied data treatments included:

• Sub-setting the initial data “CombFDICBankPanel” to keep only the few variables discussed in the present article;
• Creating three new financial ratios: (TPIMATOTr, depiR, and depdomR); where “TPIMATOTr” is the ratio of managed assets in fiduciary accounts to total assets; “depiR” is the share of interest bearing deposits in total deposits; and “depdomR” is the share of domestic deposits in total deposits;
• Defining the qualitative variables as factors and saving the resulting data into the R data object “CombFDICBankPanelB”;
• Adjusting the bank's data “CombFDICBankPanelB” for compatibility with U.S. state and county level geospatial data, which we extract from the GADM database of Global Administrative Areas [5]. The adjusted data is then saved into the R data object “CombFDICBankPanelB2”;
• Incorporating descriptive labels for the generically named variables in the adjusted Panel Data "CombFDICBankPanelB2", and saving the final result into the R data object “CombFDICBankPanelC”.

“CombFDICBankPanelC” is the final sample with 16209 observations on 5403 U.S. FDIC insured banks observed over a period of three years. From a statistical sampling standpoint, this sample is the subset of all U.S. FDIC insured financial institutions with end of year (4th quarter) financial reporting for each of the 3 fiscal years 2016, 2017 and 2018. The sampling frame is the subset of insured institutions that responded to the reporting calls during the fiscal years 2016–2018, while the target population is the set of all U.S. FDIC insured financial institutions in 2016, 2017, and 2018. The characteristics of the sampling process can therefore be summarized as follows:

• Sampling frame: 17016 (with 2016–5922, 2017–5679, 2018–5415)
• Sample size: 16209 (with 5403 observations in each year 2016, 2017, and 2018)
• Retention rate = (Sampling frame/Sample size)*100 = 95.2574%

**Fig. 1:**
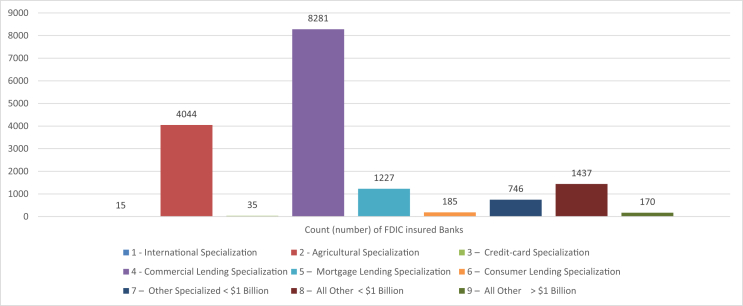
Frequency Histogram of FDIC banks across Asset Concentration Hierarchy in the Panel data “CombFDICBankPanelC”.

**Fig. 2:**
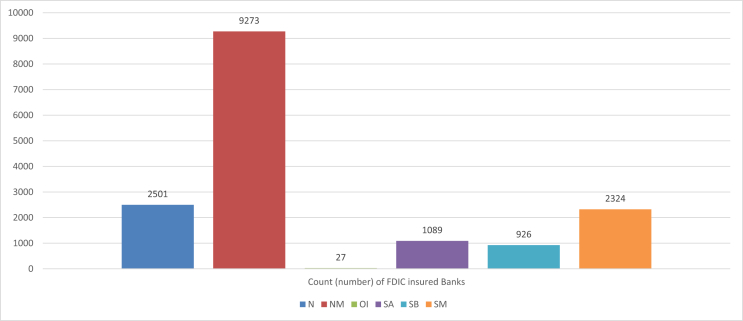
Frequency Histogram of FDIC banks across Bank Charter Class in the Panel data “CombFDICBankPanelC”.

The key experimental factors in the data are described in Table 1 and include: (i) FDIC insured banks demographic information, further summarized through the histogram representations in Fig. 1 for banks' distribution across asset concentration hierarchy and Fig. 2 for banks’ distribution across bank charter class. The remaining experimental factors in Table 1 include (ii) Performance and Condition measures, and (iii) Annual Fiduciary Activities.

**Table 1:** Variable description.

Variable	Type	Description
Year	Numeric	Year of data collection: 2016, 2017, 2018
Cert	Numeric	Institution's unique FDIC Number
Name	Character	Institution's name
webaddr	Character	Primary internet web address
City	Character	Institution headquarters city location
Zip	Numeric	zip code of the Institution headquarters location
NAME_2	Character	US county of institution's headquarter location
Stalp	Character	Institution headquarters State name abbreviation
fdicdbs	Categorical	Indicator for the Six FDIC Regions • **New York (02)-States**: Connecticut, Delaware, Maine, Maryland, Massachusetts, New Hampshire, New Jersey, New York, Pennsylvania, Puerto Rico, Rhode Island, Vermont, Virgin Islands • **Atlanta (05)-States**: Alabama, Florida, Georgia, North Carolina, South Carolina, Virginia • Chicago (09)-States: Illinois, Indiana, Kentucky, Michigan, Ohio • **Kansas City (11)** - States: Iowa, Kansas, Minnesota, Missouri, Nebraska, North Dakota, South Dakota • **Dallas (13)- States**: Arkansas, Colorado, Louisiana, Mississippi, New Mexico, Oklahoma, Tennessee, Texas • **San Francisco (14) - States**: Alaska, American Samoa, Arizona, California, Federated States of Micronesia, Guam, Hawaii, Idaho, Montana, Nevada, Oregon, Utah, Washington, Wyoming
Repdte	Date	Date of financial reporting
bkclass	Categorical	Bank Charter Class - A classification code assigned by the FDIC based on the institution's charter type (commercial bank or savings institution), charter agent (state or federal), Federal Reserve membership status (Fed member, Fed nonmember)and its primary federal regulator (state chartered institutions are subject to both federal and state supervision). • **N** = commercial bank, national (federal) charter and Fed member, supervised by the Office of the Comptroller of the Currency (OCC) • **SM** = commercial or savings bank, state charter and Fed member, supervised by the Federal Reserve (FRB) • **NM** = commercial bank, state charter and Fed nonmember, supervised by the FDIC or OCC • **SB** = savings banks, state charter, supervised by the FDIC • **SA** = As of July 21, 2011, FDIC supervised state chartered thrifts and OCC supervised federally chartered thrifts. Prior to that date, state or federally chartered savings associations supervised by the Office of Thrift Supervision (OTS). • **OI** = insured U.S. branch of a foreign chartered institution (IBA)
specgrp	Categorical	Asset Concentration Hierarchy- An indicator of an institution's primary specialization in terms of asset concentration: 1 **International Specialization**: Institutions with assets greater than $10 billion and more than 25% of total assets in foreign offices. 2 **Agricultural Specialization**: Banks with agricultural production loans plus real estate loans secured by farmland in excess of 25% of total loans and leases. 3 **Credit-card Specialization**: Institutions with credit-card loans plus securitized receivables in excess of 50% of total assets plus securitized receivables. 4 **Commercial Lending Specialization**: Institutions with commercial and industrial loans, plus real estate construction and development loans, plus loans secured by commercial real estate properties in excess of 25% of total assets. 5 **Mortgage Lending Specialization**: Institutions with residential mortgage loans, plus mortgage-backed securities, in excess of 50% of total assets. 6 **Consumer Lending Specialization**: Institutions with residential mortgage loans, plus credit-card loans, plus other loans to individuals, in excess of 50% of total assets. 7 **Other Specialized < $1 Billion**: Institutions with assets less than $1 billion and with loans and leases are less than 40% of total assets. 8 **All Other < $1 Billion**: Institutions with assets less than $1 billion that do not meet any of the definitions above, they have significant lending activity with no identified asset concentrations. 9 **All Other > $1 Billion**: Institutions with assets greater than $1 billion that do not meet any of the definitions above, they have significant lending activity with no identified asset concentrations.
offdom	Integer	Number of Domestic U.S. Offices (including headquarters) in the 50 states of the U.S.A. operated by the FDIC Insured institution
Offfor	Integer	Number of Foreign Offices (outside the U.S. and U.S. territories) operated by the active FDIC Insured institution
stmult	Categorical	Institution with Inter-state Branches (yes/no)
trpower	Categorical	Fiduciary powers granted (yes/no)
Trexer	Categorical	Fiduciary power exercised (yes/no)
TPIMATOT	Numeric	Total managed assets in fiduciary account ($)
numemp	Integer	Total employees (full-time equivalent)
Asset	Numeric	Total assets ($)
Dep	Numeric	Total deposits ($)
Depi	Numeric	Interest-bearing deposits ($)
depdom	Numeric	Deposits held in domestic offices ($)
intincy	Ratio	Yield on earning assets- Total interest income (annualized) as a percent of the average of all loans and other investments that earn interest or dividends (Average Earning Assets). Average earning assets in year-end quarter 4 (December) is calculated as: (Previous December earning assets + March earning assets + June earning assets + September earning assets + December earning assets)/5
Roa	Ratio	Return on assets - Net income after taxes and extraordinary items (annualized) as a percent of average total assets.
Roe	Ratio	Return on Equity - Annualized net income as a percent of average total equity on a consolidated basis. Note: If retained earnings are negative, the ratio is shown as “NA”.
Eeffr	Ratio	Efficiency ratio - Noninterest expense less amortization of intangible assets as a percent of net interest income plus noninterest income. This ratio measures the proportion of net operating revenues that are absorbed by overhead expenses, so that a lower value indicates greater efficiency.
rbc1aaj	Ratio	Core capital (leverage) ratio - Tier 1 (core) capital as a percent of average total assets minus ineligible intangibles. Tier 1 (core) capital includes: common equity plus noncumulative perpetual preferred stock plus minority interests in consolidated subsidiaries less goodwill and other ineligible intangible assets. The amount of eligible intangibles (including mortgage servicing rights) included in core capital is limited in accordance with supervisory capital regulations. Average total assets used in this computation are an average of daily or weekly figures for the quarter.
rbc1rwaj	Ratio	Tier 1 risk-based capital ratio - Tier 1 (core) capital as a percent of risk-weighted assets as defined by federal regulators for prompt corrective action during a time period.
rbcrwaj	Ratio	Total risk-based capital ratio - Total risk based capital as a percent of risk-weighted assets as defined by federal regulators for prompt corrective action during a time period.
Nimy	Ratio	Net interest margin - Total interest income less total interest expense (annualized) as a percent of the average of all loans and other investments that earn interest or dividends (Average Earning Assets).
Noijy	Ratio	Net operating income to assets - Net operating income (annualized) as a percent of the Year-to-date average of the total assets represented on the balance sheet (average total assets). Average total assets in year-end quarter 4 (December) is calculated as: (Previous December earning assets + March earning assets + June earning assets + September earning assets + December earning assets)/5
ntlnlsr	Ratio	Net charge-offs to loans - Gross loan and lease financing receivable charge-offs, less gross recoveries, (annualized) as a percent of average total loans and lease financing receivables. Average total loans in year-end quarter 4 (December) is calculated as: (Previous December total loans + March total loans + June total loans + September total loans + December total loans)/5
elnantr	Ratio	Credit loss provision to net charge-offs - Provision for possible credit and allocated transfer risk as a percent of net charge-offs. If the denominator is less than or equal to zero, then ratio is shown as "NA."
ernastr	Ratio	Earning assets to total assets ratio - Income before income taxes and extraordinary items and other adjustments, plus provisions for loan and lease losses and allocated transfer risk reserve, plus gains (losses) on securities not held in trading accounts (annualized) divided by net loan and lease charge-offs (annualized). This is a number of times ratio (x) not a percentage ratio (%). If the denominator is less than or equal to zero, then ratio is shown as "NA."
astempm	Ratio	Assets per employee ($millions) - Total assets in millions of dollars as a percent of the number of full-time equivalent employees.
lnlsntv	Ratio	Net loans and leases to total assets - Loan and lease financing receivables, net of unearned income, allowances, and reserves, as a percent of total assets.
depdastr	Ratio	Total domestic deposits to total assets - Total domestic office deposits as a percent of total assets.
Eqv	Ratio	Equity capital to assets - Total equity capital as a percent of total assets.
asset5	Numeric	Year-to-date Average total assets ($) - Year-to-date average of the total assets represented on the institution's balance sheet.
ernast5	Numeric	Year-to-date Average earning assets ($) - The average of all loans and other investments that earn interest or dividends.
eq5	Numeric	Year-to-date Average equity ($) - The average of total equity capital (includes preferred and common stock, surplus and undivided profits).
lnlsgr5	Numeric	Year-to-date Average total loans ($) - The average of total loans and lease financing receivables, net of unearned income.
iddepinr	Ratio	Percent of Insured deposits in total deposit liabilities - Estimated amount of insured deposits as a percent of total deposit liabilities before exclusions (gross) as defined in section 3(l) of the Federal Deposit Insurance Act and FDIC regulations.
TPIMATOTr	Ratio	Ratio of Total assets in fiduciary account to total assets
depiR	Ratio	Ratio of interest bearing deposits to total assets
depdomR	Ratio	Ratio of Domestic deposits to total assets
NAME_1	Character	Institution headquarters State full name

**Table 2:** Means and standard deviation of Aggregated FDIC banks performance measures across years of reporting.

Year		2016	2017	2018
Count (number) of FDIC insured Banks		5380	5380	5380
Average performance measures	BkAvrg**Asset5**	2978558	3120466	3257891
	BkSd**Asset5**	(48346876)	(50087037)	(50866987)
	BkAvrg**Ernast5**	2663585	2805648	2937984
	BkSd**Ernast5**	(42813606)	(44679941)	(45453709)
	BkAvrg**Eq5**	331813.5	348445.7	365145.7
	BkSd**Eq5**	(5153870)	(5263982)	(5336279)
	BkAvrg**Lnlsgr5**	1640645	1728929	1835701
	BkSd**Lnlsgr5**	(23376407)	(24014535)	(24638892)
	BkAvrg**Astempm**	6.141	6.416	6.495
	BkSd**Astempm**	(25.008)	(27.106)	(24.026)
	BkAvrg**Roa**	1.061	1.026	1.225
	BkSd**Roa**	(3.534)	(4.078)	(5.589)
	BkAvrg**Roe**	8.596	8.305	9.811
	BkSd**Roe**	(11.773)	(13.499)	(15.421)
	BkAvrg**Eeffr**	70.760	68.911	67.612
	BkSd**Eeffr**	(34.036)	(29.110)	(47.806)
	BkAvrg**Rbc1aaj**	11.708	11.841	12.144
	BkSd**Rbc1aaj**	(6.733)	(6.759)	(6.713)
	BkAvrg**Rbc1rwaj**	25.918	26.144	20.748
	BkSd**Rbc1rwaj**	(476.893)	(465.037)	(82.150)
	BkAvrg**Rbcrwaj**	27.003	27.213	21.804
	BkSd**Rbcrwaj**	(476.874)	(465.017)	(82.114)

Note: The information tabulated here is extracted from the R data frame “yearOutcomeDat” in the attached supplementary material, which was produced by splitting the shared data “CombFDICBankPanelC” into its cross-sectional planes using “dplyr” [6], and then computing the mean and standard deviations (shown in parenthesis) for a select key performance measures. For each performance measure, the name of the data object capturing its computed mean in “yearOutcomeDat” is preceded by “BkAvrg” followed by the name of the variable itself as in the R data file “CombFDICBankPanelC”. Similarly, the name of the data object capturing its computed standard deviation in “yearOutcomeDat” is preceded by “BkSd” followed by the name of the variable itself as in the R data file “CombFDICBankPanelC”. (For more details, see the attached R computer codes).

**Table 3:** Means and standard deviation of Aggregated FDIC banks performance measures across Asset Concentration Hierarchy.

Asset Concentration Hierarchy		1 - International Specialization	2 - Agricultural Specialization	3 –Credit-card Specialization	4 - Commercial Lending Specialization	5 –Mortgage Lending Specialization	6 –Consumer Lending Specialization	7 –Other Specialized < $1 Billion	8 –All Other < $1 Billion	9 –All Other > $1 Billion
Count (number) of FDIC insured Banks		15	4044	35	8281	1227	185	746	1437	170
Average performance measures	SgAvrg**Asset5**	829210166.8	20333.8	47046437	2056432.7	750538.9	3843868.6	172131.2	175653.5	96417478.9
	SgSd**Asset5**	(815092587.3)	(499710.6)	(48889017)	(12303946.5)	(3180579.1)	(13439545)	(248261.4)	(156302.8)	(321326330)
	SgAvrg**Ernast5**	726555876.2	189518.8	43353545.1	1846401.7	711560.6	3735857.3	159817.2	163084.2	90730895.1
	SgSd**Ernast5**	(720735374.9)	(461769.6)	(44125827.2)	(10954068.4)	(3040731)	(13096342)	(236291.2)	(145447.4)	(294469737.3)
	SgAvrg**Eq5**	82185862.68	23132.22	7139597.79	242473.49	85915.34	388056.57	26852.89	20907.83	11089839.86
	SgSd**Eq5**	(82961893.02)	(61781.52)	(9109797.39)	(1477741.31)	(314864.94)	(1279300.51)	(47066.58)	(22969.54)	(35570913.84)
	SgAvrg**Lnlsgr5**	303109496.60	137765.22	37143873.44	1429830.45	461855.55	2719556.78	56141.35	97777.76	50929190.42
	SgSd**Lnlsgr5**	(353718563.1)	(368998.5)	(38447593.9)	(8179059.1)	(1881543.1)	(9152486)	(134190.6)	(89999.2)	(176036550.1)
	SgAvrg**Astempm**	8.292	5.159	45.314	5.628	10.964	13.119	5.916	4.379	41.041
	SgSd**Astempm**	(1.650)	(1.876)	(90.152)	(4.357)	(80.804)	(42.177)	(12.855)	(1.966)	(86.782)
	SgAvrg**Roa**	0.922	1.100	2.506	0.988	0.852	0.856	3.213	0.864	1.446
	SgSd**Roa**	(0.271)	(0.607)	(1.472)	(0.808)	(2.486)	(0.935)	(20.260)	(0.730)	(1.814)
	SgAvrg**Roe**	10.330	9.716	17.485	9.018	5.411	7.131	10.744	7.594	12.469
	SgSd**Roe**	(3.292)	(5.849)	(10.834)	(7.786)	(6.672)	(8.077)	(54.093)	(7.005)	(12.952)
	SgAvrg**Eeffr**	64.802	65.383	46.675	69.349	77.856	67.812	67.292	73.552	57.864
	SgSd**Eeffr**	(7.068)	(13.448)	(13.978)	(23.638)	(18.603)	(20.509)	(149.336)	(16.306)	(19.956)
	SgAvrg**Rbc1aaj**	8.044	11.572	14.396	10.898	14.893	12.356	19.432	12.092	11.319
	SgSd**Rbc1aaj**	(0.867)	(3.143)	(3.707)	(3.072)	(10.978)	(6.023)	(20.865)	(6.500)	(8.703)
	SgAvrg**Rbc1rwaj**	13.968	17.551	15.497	14.478	33.503	18.399	155.457	22.361	32.594
	SgSd**Rbc1rwaj**	(1.704)	(7.965)	(4.065)	(4.956)	(51.461)	(8.708)	(1798.174)	(23.492)	(91.767)
	SgAvrg**Rbcrwaj**	15.167	18.634	17.115	15.841	34.447	19.456	156.360	23.444	33.595
	SgSd**Rbcrwaj**	(1.506)	(7.960)	(3.885)	(4.973)	(51.338)	(8.713)	(1798.113)	(23.444)	(91.616)

Note: The information tabulated here is extracted from the R data frame “specgrpOutcomeDat” in the attached supplementary material, which was produced by splitting the shared data “CombFDICBankPanelC” into its planes formed by banks' primary area of specialization, using “dplyr” [6], and then computing the mean and standard deviations (shown in parenthesis) for the select key performance measures. (See the attached R computer codes, for more details).

**Table 4:** Means and standard deviation of Aggregated FDIC banks performance measures across Bank Charter Class.

Bank Charter Class		N	NM	OI	SA	SB	SM
Count (number) of FDIC insured Banks		2501	9273	27	1089	926	2324
Average performance measures	SgAvrg**Asset5**	12938081.9	736529.2	10303263.9	1841887.9	1205220.8	3335709.4
	SgSd**Asset5**	(736529.2)	(5205943)	(16714341)	(11673572)	(3840832)	(19852134)
	SgAvrg**Ernast5**	11590527	678816.7	NA	1766181.7	1115937.7	2987228.6
	SgSd**Ernast5**	(110251647)	(4680316)	(NA)	(11475355)	(3474853)	(17408794)
	SgAvrg**Eq5**	1429797.6	87841.51	NA	186062.49	145578.61	381654.17
	SgSd**Eq5**	(13024005.4)	(660201.5)	(NA)	(969246.9)	(474417.7)	(2215407.5)
	SgAvrg**Lnlsgr5**	6896869.6	519031.3	5212410.7	960230.3	883670.1	1741530.8
	SgSd**Lnlsgr5**	(59751236)	(3655092)	(6108378)	(4714218)	(2821678)	(9405828)
	SgAvrg**Astempm**	8.413	5.563	NA	8.369	6.857	6.134
	SgSd**Astempm**	(54.920)	(8.863)	(NA)	(42.142)	(6.125)	(8.381)
	SgAvrg**Roa**	1.151	1.062	NA	1.921	0.607	1.036
	SgSd**Roa**	(2.405)	(1.104)	(NA)	(16.503)	(0.540)	(0.620)
	SgAvrg**Roe**	9.057	9.343	NA	7.178	5.099	9.311
	SgSd**Roe**	(7.340)	(7.963)	(NA)	(44.207)	(4.216)	(7.262)
	SgAvrg**Eeffr**	67.788	68.068	NA	79.602	76.187	66.845
	SgSd**Eeffr**	(46.198)	(41.573)	(NA)	(23.626)	(14.512)	(17.556)
	SgAvrg**Rbc1aaj**	12.202	11.410	NA	16.711	12.484	11.025
	SgSd**Rbc1aaj**	(9.212)	(4.849)	(NA)	(14.144)	(4.382)	(3.930)
	SgAvrg**Rbc1rwaj**	51.578	17.548	NA	37.689	20.883	16.767
	SgSd**Rbc1rwaj**	(981.034)	(17.349)	(NA)	(77.577)	(12.678)	(18.025)
	SgAvrg**Rbcrwaj**	52.677	18.620	NA	38.648	21.911	17.860
	SgSd**Rbcrwaj**	(980.996)	(17.311)	(NA)	(77.435)	(12.708)	(17.986)

Note: The information tabulated here is extracted from the R data frame “bkclassOutcomeDat”in the attached supplementary material, which was produced by splitting the shared data “CombFDICBankPanelC” into its planes formed by banks' charter class, using “dplyr” [6], and then computing the mean and standard deviations (shown in parenthesis) for the select key performance measures. (See the attached R computer codes, for more details).

**Fig. 3:**
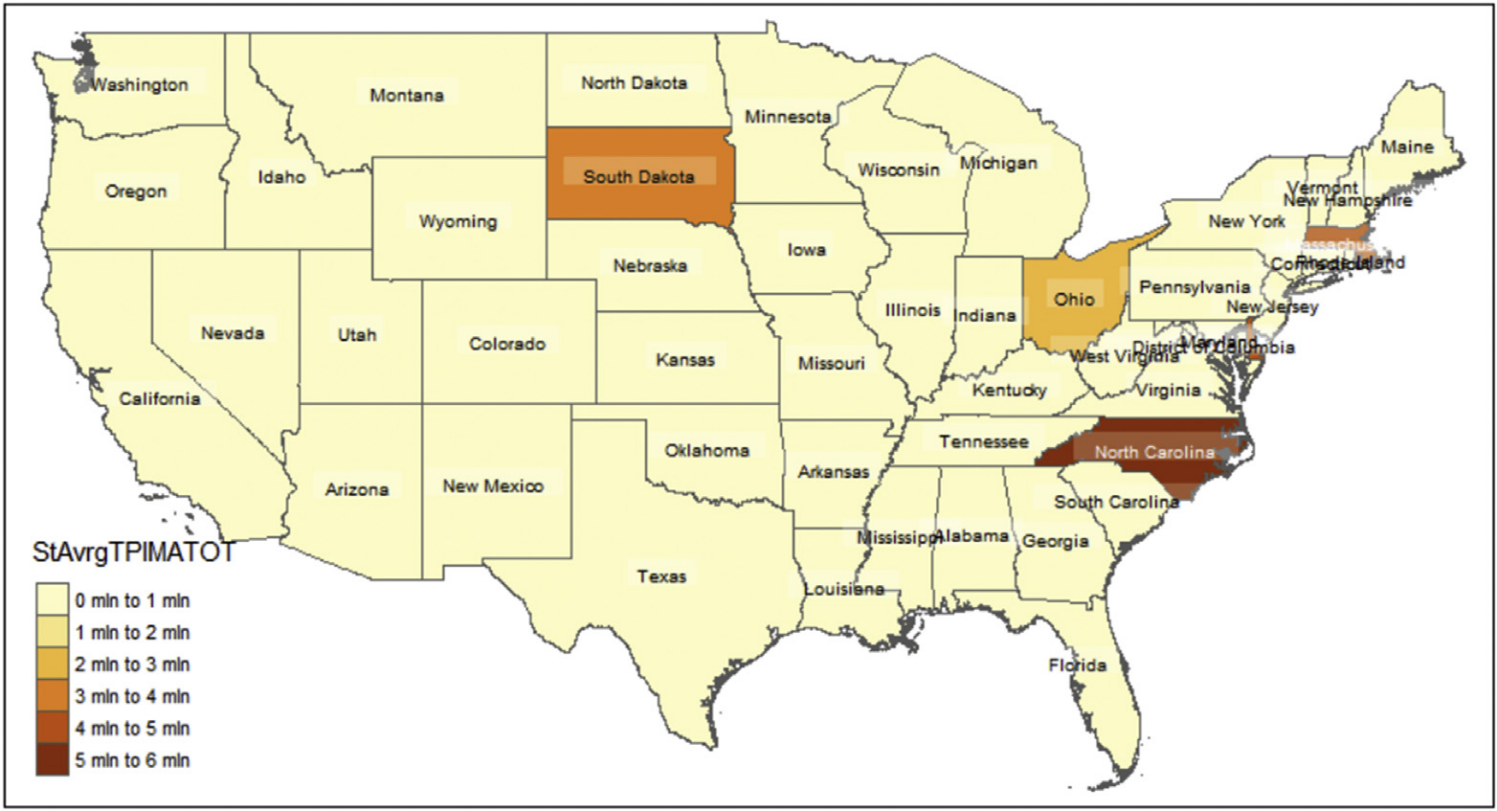
Distribution of Panel wide (2016–2018) state level average of "Total managed assets in fiduciary account ($)".

**Fig. 4:**
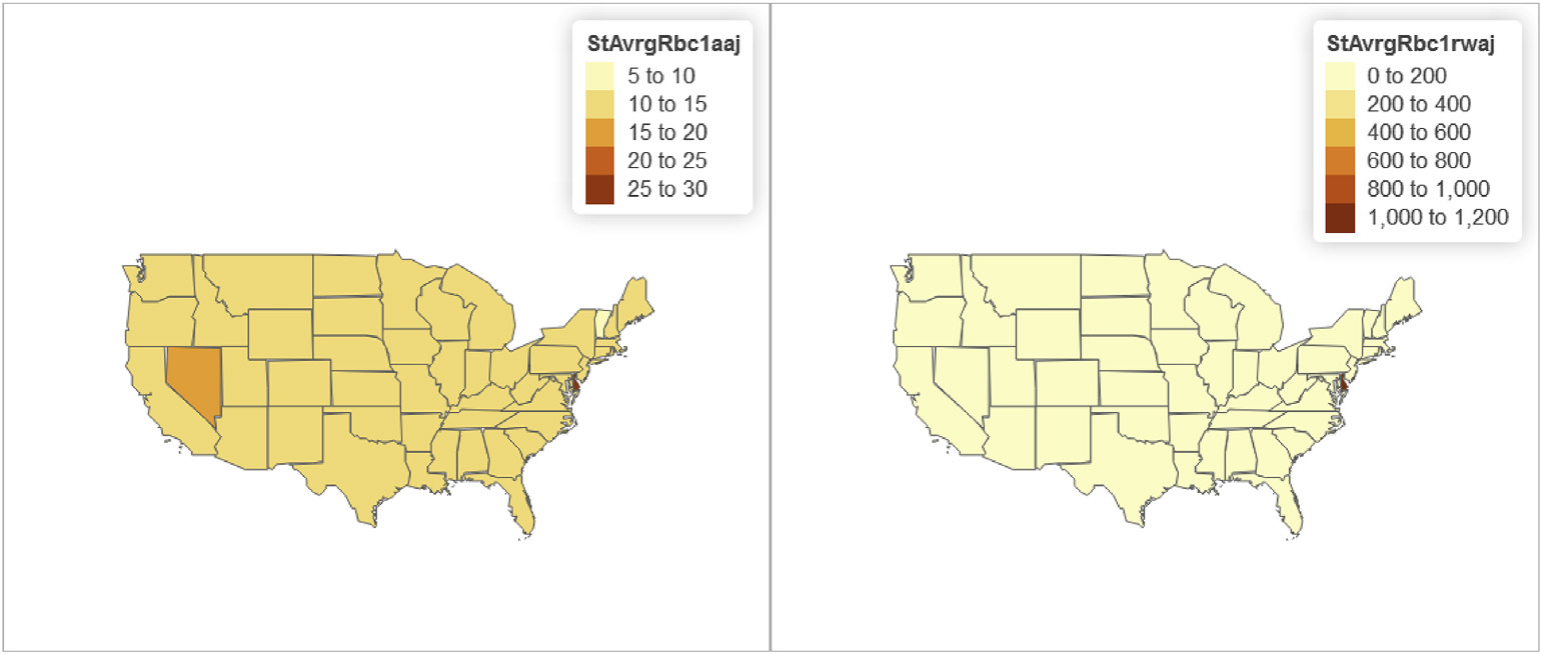
Panel wide (2016–2018) state level distribution of "Core capital (leverage) ratio” and "Tier 1 risk-based capital ratio".

**Fig. 5:**
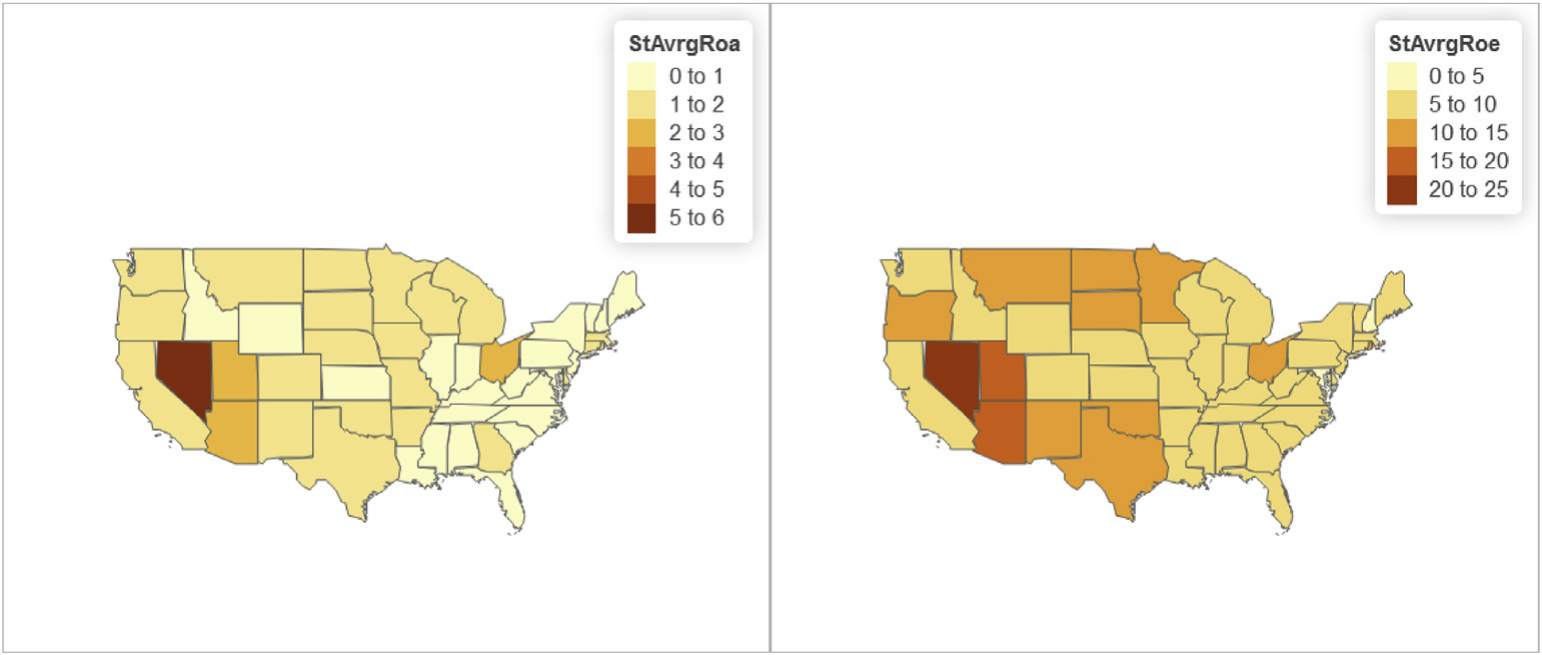
Panel wide (2016–2018) state level distributions of "Return on Assets - ROA” and "Return on Equity - ROE".

**Fig. 6:**
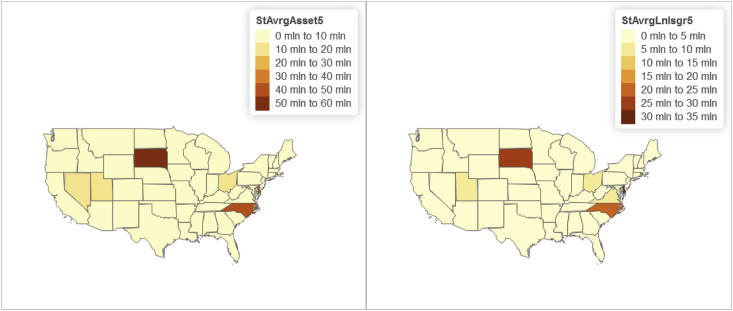
Panel wide (2016–2018) state level distributions of year-to-date "Average Total Assets” and "Average Total Loans".

**Fig. 7:**
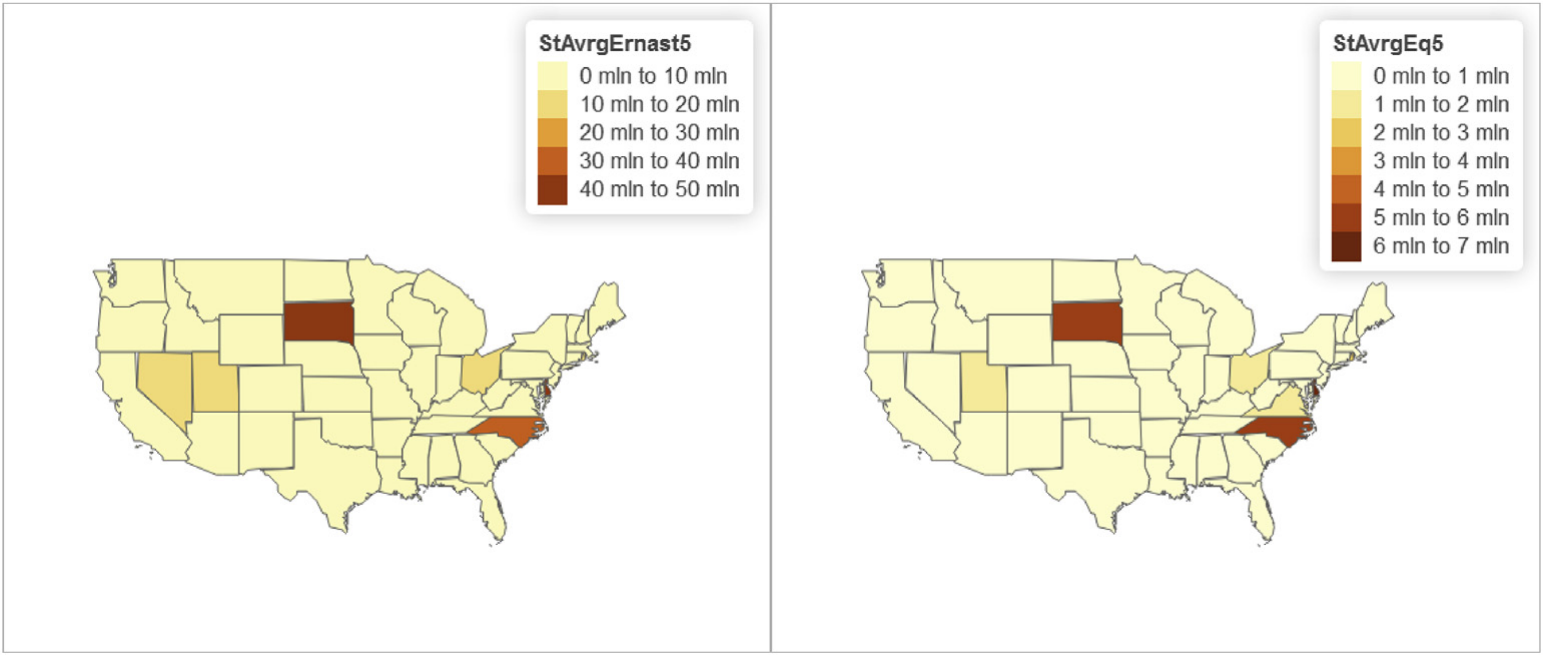
Panel wide (2016–2018) state level distributions of year-to-date "Average earning assets ($)” and "Average equity ($)".

In addition to the description of the variables in Table 1, using the R library “dplyr” [6], we provide descriptive analytics of FDIC banks performances across key banks’ demographic characteristics including:

• Years of reporting [see Table 2];
• Asset concentration Hierarchy [see Table 3];
• Bank charter class [see Table 4].

Furthermore relying on the U.S. State-level geospatial information from our constructed R data frame “statesOutcomeDat1”, we also map aggregated key variables including the Panel wide (2016–2018) state level average of:

• Total managed assets in fiduciary account ($) shown in Fig. (3);
• “Core capital (leverage) ratio” and "Tier 1 risk-based capital ratio” shown in Fig. (4);
• “ROE” and "ROA” shown in Fig. (5);
• Year-to-date "average total assets” and "average total loans” shown in Fig. (6);
• Year-to-date "average earning assets ($)” and "average equity ($)” shown in Fig. (7);
