# Supplementary material for: Panel and geospatial data for U.S. FDIC insured banks fiduciary activities and annual performance analyses over the periods 2016 to 2018
Source: Data Brief. 2019 Aug 6;25:104358. doi: 10.1016/j.dib.2019.104358 (PMC6704373; doi:10.1016/j.dib.2019.104358)
Supplement: Supplementary file 1 [file mmc1.zip › R data and Codes Supplements/0-Initial Panel data/Raw Excel Format FDIC banks reporting Data/All_Reports_20181231_readme.htm]

FDIC: Custom Form Selection


|  |  |
| --- | --- |
| **Large Download ReadMe File** | **Requested: 2/19/2019** |
|  | **Created:      2/19/2019** |

|  |  |  |
| --- | --- | --- |
| **Search Criteria** | | |
| Institution Status | | Active |
| Report Date | | 12/31/2018 |
| Sort Order | | Order by NAME |
|  | | |
| **List of Variables in Download Files** | | |
| **Assets and Liabilities** | | |
| **Definition** | **Name** | **Label** |
| 1 | numemp | Total employees (full-time equivalent) |
| 2 | asset | Total assets |
| 3 | chbal | Cash & Balances due from depository institutions |
| 4 | chbali | Interest-bearing balances |
| 5 | sc | Total securities |
| 6 | frepo | Federal funds sold and reverse repurchase |
| 7 | lnlsnet | Net loans and leases |
| 8 | lnatres | Loan loss allowance |
| 9 | trade | Trading account assets |
| 10 | bkprem | Bank premises and fixed assets |
| 11 | ore | Other real estate owned |
| 12 | intan | Goodwill and other intangibles |
| 13 | idoa | All other assets |
| 14 | liabeq | Total liabilities and capital |
| 15 | liab | Total Liabilities |
| 16 | dep | Total deposits |
| 17 | depi | Interest-bearing deposits |
| 18 | depdom | Deposits held in domestic offices |
| 19 | iddepinr | % insured (estimated) |
| 20 | frepp | Federal funds purchased and repurchase agreements |
| 21 | tradel | Trading liabilities |
| 22 | idobrmtg | Other borrowed funds |
| 23 | subnd | Subordinated debt |
| 24 | idoliab | All other liabilities |
| 25 | eqtot | Total equity capital |
| 26 | eq | Bank equity capital |
| 27 | eqpp | Perpetual preferred stock |
| 28 | eqcs | Common stock |
| 29 | eqsur | Surplus |
| 30 | equptot | Undivided profits |
| 31 | eqconsub | Equity, minor interest in consolidated subs |
| 32 | nclnls | Noncurrent loans and leases |
| 33 | ncgtypar | Noncurrent loans which are wholly or partially guaranteed by the U.S. |
| 34 | oaienc | Income earned, not collected on loans |
| 35 | ernast | Earning assets |
| 36 | asstlt | Long-term assets (5+ years) |
| 37 | Asset5 | Average total assets |
| 38 | asset2 | Average assets, quarterly |
| 39 | RWAJT | Total risk weighted assets adjusted |
| 40 | avassetj | Adjusted average assets for leverage capital purposes |
| 41 | OALIFINS | Life insurance assets |
| 42 | OALIFGEN | General account life insurance assets |
| 43 | OALIFSEP | Separate account life insurance assets |
| 44 | Oalifhyb | Hybrid life insurance assets |
| 45 | voliab | Volatile liabilities |
| 46 | lnexamt | Insider loans |
| 47 | othbfhlb | FHLB advances |
| 48 | lnlssale | Loans and leases held for sale |
| 49 | ucln | Unused loan commitments |
| 50 | rbct1j | Tier one (core) capital |
| 51 | rbct2 | Tier 2 Risk-based capital |
| 52 | uc | Total unused commitments |
| 53 | obsdir | Derivatives |
| **Cash and Balances Due** | | |
| **Definition** | **Name** | **Label** |
| 1 | chbal | Cash & Balances due from depository institutions |
| 2 | chcic | Cash items in process of collection |
| 3 | chitem | Collection in domestic offices |
| 4 | chcoin | Currency and coin in domestic offices |
| 5 | chus | Balances due from depository institutions in U.S. |
| 6 | chusfbk | U.S. branches of foreign banks |
| 7 | chnus | Balances due from foreign banks |
| 8 | chnusfbk | Foreign branches of U.S. banks |
| 9 | chfrb | Balances due from FRB |
| 10 | chbalni | Total noninterest-bearing balances |
| **Securities** | | |
| **Definition** | **Name** | **Label** |
| 1 | sc | Total securities |
| 2 | scus | U.S. Government securities |
| 3 | scust | U.S. Treasury securities |
| 4 | scage | U.S. Government agency obligations |
| 5 | scmuni | Securities issued by states & political subdivisions |
| 6 | scdomo | Other domestic debt securities |
| 7 | IDSCOD | Privately issued residential mortgage-backed securities |
| 8 | SCCMMB | Commercial mortgage-backed securities - Total |
| 9 | scabs | Asset backed securities |
| 10 | SCSFP | Structured financial products - Total |
| 11 | SCODOT | Other domestic debt securities - All other |
| 12 | scford | Foreign debt securities |
| 13 | IDsceq | Equity securities not held for trading |
| 14 | sceq | Equity securities available-for-sale |
| 15 | sceqfv | Equity securities readily determinable fair values |
| 16 | idahta | Assets held in trading accounts for TFR Reporters |
| 17 | scres | General valuation allowances for securities for TFR Reporters |
| 18 | scpledge | Pledged securities |
| 19 | scmtgbk | Mortgage-backed securities |
| 20 | idscgtpc | Certificates of participation in pools of residential mortgages |
| 21 | scgty | Issued or guaranteed by U.S. |
| 22 | scodpc | Privately issued |
| 23 | idsccmo | Collaterized mortgage obligations |
| 24 | sccol | CMOs issued by government agencies or sponsored agencies |
| 25 | scodpi | Privately issued |
| 26 | IDSCCMT | Commercial mortgage-backed securities |
| 27 | SCCMPT | Commercial mortgage pass-through securities |
| 28 | SCCMOT | Other commercial mortgage-backed securities |
| 29 | scha | Held to maturity securities (book value) |
| 30 | scaf | Available-for-sale securities (fair market value) |
| 31 | scrdebt | Total debt securities |
| 32 | scsnhaa | Amortized cost |
| 33 | scsnhaf | Fair value |
| 34 | trade | Trading account assets |
| 35 | idtrrval | Revaluation gains on off-balance sheet contracts |
| 36 | trlreval | Revaluation losses on off-balance sheet contracts |
| **U.S. Government Obligations** | | |
| **Definition** | **Name** | **Label** |
| 1 | scage | U.S. Government agency obligations |
| 2 | IDscas | Non-mortgage backs issued by U.S. government agencies and by U.S. GSEs |
| 3 | scaot | U.S. government agencies |
| 4 | scspn | Issued by U.S. government enterprises (GSEs) |
| 5 | scfmn | Issued by F.N.M.A. and F.H.L.M.C. |
| 6 | scgnm | Issued by G.N.M.A. |
| 7 | sccol | CMOs issued by government agencies or sponsored agencies |
| 8 | SCCPTG | Commercial mortgage pass-through securities � FNMA, FHLMC, or GNMA |
| 9 | SCCMOG | Other commercial MBS Issued or guaranteed by US Govrnmnt agencies or sponsored |
| **Total Debt Securities** | | |
| **Definition** | **Name** | **Label** |
| 1 | scrdebt | Total debt securities |
| 2 | scpt3les | Three months or less |
| 3 | scpt3t12 | Over three months through twelve months |
| 4 | scpt1t3 | Over one year through three years |
| 5 | scpt3t5 | Over three years through five years |
| 6 | scpt5t15 | Over five years through fifteen years |
| 7 | scptov15 | Over fifteen years |
| 8 | sco3yles | Three years or less |
| 9 | scoov3y | Over three years |
| 10 | scnm3les | Three months or less |
| 11 | scnm3t12 | Over three months through twelve months |
| 12 | scnm1t3 | Over one year through three years |
| 13 | scnm3t5 | Over three years through five years |
| 14 | scnm5t15 | Over five years through fifteen years |
| 15 | scnmov15 | Over fifteen years |
| 16 | sc1les | With remaining maturity of one year or less |
| **Net Loans and Leases** | | |
| **Definition** | **Name** | **Label** |
| 1 | lnlsnet | Net loans and leases |
| 2 | lnatres | Loan loss allowance |
| 3 | lnlsgr | Total loans and leases |
| 4 | lncontra | Unearned income |
| 5 | idlnls | Loans and leases, gross |
| 6 | lnre | All real estate loans |
| 7 | lnredom | Real estate loans in domestic offices |
| 8 | lnrecons | Construction and development loans |
| 9 | LNRECNFM | Residential 1-4 family construction |
| 10 | LNRECNOT | Other construction, all land development and other land |
| 11 | lnrenres | Secured by nonfarm nonresidential properties |
| 12 | LNRENROW | Nonfarm nonresidential secured by owner-occupied properties |
| 13 | LNRENROT | Commercial real estate other non-farm non-residential |
| 14 | lnremult | Multifamily residential real estate |
| 15 | lnreres | 1-4 family residential loans |
| 16 | lnreag | Farmland loans |
| 17 | lnrefor | Loans held in foreign offices |
| 18 | lnag | Farm loans |
| 19 | lnci | Commercial and industrial loans |
| 20 | lncinus | To non-U.S. addressees |
| 21 | lncon | Loans to individuals |
| 22 | lncrcd | Credit card loans |
| 23 | lnconrp | Related Plans |
| 24 | LNAUTO | Consumer Loans - Auto |
| 25 | lnconoth | Other loans to individuals |
| 26 | lnotci | All other loans & leases |
| 27 | lnfg | Loans to foreign governments and official institutions |
| 28 | lnmuni | Obligations of states and political subdivisions in U.S. |
| 29 | idothlns | Other loans |
| 30 | ls | Lease financing receivables |
| 31 | lndepac | Loans to depository institutions and acceptances of other banks |
| 32 | lncomre | Loans not secured by real estate |
| 33 | lnrenus | Loans secured by real estate to non-U.S. addressees |
| 34 | rslnltot | Restructured Loans & leases |
| 35 | rslnls | Non 1-4 family restructured loans & leases |
| 36 | RB2LNRES | Allowance for loan and lease losses in tier 2 |
| 37 | lnlsgrf | Total loans and leases |
| **1-4 Family Residential Net Loans and Leases** | | |
| **Definition** | **Name** | **Label** |
| 1 | lnreres | 1-4 family residential loans |
| 2 | lnrersfm | Loans secured by 1-4 family first liens |
| 3 | lnrersf2 | Loans secured by 1-4 family junior liens |
| 4 | lnreloc | Home equity loans |
| 5 | LNRERSF1 | Adjustable rate loans secured by 1-4 family residential (memoranda) |
| **Loans to Depository Institutions** | | |
| **Definition** | **Name** | **Label** |
| 1 | lndepac | Loans to depository institutions and acceptances of other banks |
| 2 | lndepcb | To commercial banks in U.S. |
| 3 | lndepusb | To U.S. branches and agencies of foreign banks |
| 4 | lndepus | To other depository institutions in U.S. |
| 5 | lndepfc | To banks in foreign countries |
| 6 | lndepfus | To foreign branches of U.S. banks |
| **Total Loans and Leases in Foreign Offices** | | |
| **Definition** | **Name** | **Label** |
| 1 | lnlsgrf | Total loans and leases |
| 2 | unincfor | Unearned income |
| 3 | lnlsfor | Loans and leases, gross |
| 4 | lnrefor | Loans held in foreign offices |
| 5 | idlndacf | Loans to Depository Institutions & Acceptances of Other Banks � Foreign offices |
| 6 | lndepcbf | To commercial banks in U.S. |
| 7 | lndepusf | To other depository institutions in U.S. |
| 8 | lndepfcf | To banks in foreign countries |
| 9 | lnagfor | Farm loans |
| 10 | lncifor | Commercial and industrial loans |
| 11 | lncinusf | To non-U.S. addressees |
| 12 | lnconfor | Loans to individuals |
| 13 | lnfgfor | Loans to foreign governments and official institutions |
| 14 | lnmunif | Obligations of states and political subdivisions in U.S. |
| 15 | lnotherf | Other loans |
| 16 | lsfor | Lease financing receivables |
| **Maturity & Repricing for Loans and Leases** | | |
| **Definition** | **Name** | **Label** |
| 1 | lnls | Loans and leases, gross |
| 2 | lnrs3les | Three months or less |
| 3 | lnrs3t12 | Over three months through twelve months |
| 4 | lnrs1t3 | Over one year through three years |
| 5 | lnrs3t5 | Over three years through five years |
| 6 | lnrs5t15 | Over five years through fifteen years |
| 7 | lnrsov15 | Over fifteen years |
| 8 | lnot3les | Three months or less |
| 9 | lnot3t12 | Over three months through twelve months |
| 10 | lnot1t3 | Over one year through three years |
| 11 | lnot3t5 | Over three years through five years |
| 12 | lnot5t15 | Over five years through fifteen years |
| 13 | lnotov15 | Over fifteen years |
| **Small Business Loans** | | |
| **Definition** | **Name** | **Label** |
| 1 | Lnrenr4 | $ amt. loans sec. by nonfarm nonres. props. - orig. amts. of $1M or less |
| 2 | Lnrenr1 | $ amt. loans sec. by nonfarm nonres. props. - orig. amts. of $100K or less |
| 3 | Lnrenr2 | $ amt. loans sec. by nonfarm nonres. props. - orig. amts. $100K- $250K |
| 4 | Lnrenr3 | $ amt. loans sec. by nonfarm nonres. props. - orig. amts. $250K - $1M |
| 5 | Lnci4 | $ amt. C&I loans to U.S. addressees - orig. amts. of $1M or less |
| 6 | Lnci1 | $ amt. C&I loans to U.S. addressees - orig. amts. of $100K or less |
| 7 | Lnci2 | $ amt. C&I loans to U.S. addressees - orig. amts. $100K - $250K |
| 8 | Lnci3 | $ amt. C&I loans to U.S. addressees - orig. amts. $250K - $1M |
| 9 | Lnreag4 | $ amt. loans sec. by farmland - orig. amts. of $500K or less |
| 10 | Lnreag1 | $ amt. loans sec. by farmland - orig. amts. of $100K or less |
| 11 | Lnreag2 | $ amt. loans sec. by farmland - orig. amts. $100K - $250K |
| 12 | Lnreag3 | $ amt. loans sec. by farmland - orig. amts. $250K - $500K |
| 13 | Lnag4 | $ amt. loans to finance agricultural prod. - orig. amts. of $500K or less |
| 14 | Lnag1 | $ amt. loans to finance agricultural prod. - orig. amts. of $100K or less |
| 15 | Lnag2 | $ amt. loans to finance agricultural prod. - orig. amts. $100K - $250K |
| 16 | Lnag3 | $ amt. loans to finance agricultural prod. - orig. amts. $250K - $500K |
| 17 | Lnrenr4N | # of loans sec. by nonfarm nonres. props. - orig. amts. of $1M or less |
| 18 | Lnrenr1N | # of loans sec. by nonfarm nonres. props. - orig. amts. of $100K or less |
| 19 | Lnrenr2N | # of loans sec. by nonfarm nonres. props. - orig. amts. $100K- $250K |
| 20 | Lnrenr3N | # of loans sec. by nonfarm nonres. props. - orig. amts. $250K - $1M |
| 21 | Lnci4N | # of C&I loans to U.S. addressees - orig. amts. of $1M or less |
| 22 | Lnci1N | # of C&I loans to U.S. addressees - orig. amts. of $100K or less |
| 23 | Lnci2N | # of C&I loans to U.S. addressees - orig. amts. $100K - $250K |
| 24 | Lnci3N | # of C&I loans to U.S. addressees - orig. amts. $250K - $1M |
| 25 | Lnreag4N | # of loans sec. by farmland - orig. amts. of $500K or less |
| 26 | Lnreag1N | # of loans sec. by farmland - orig. amts. of $100K or less |
| 27 | Lnreag2N | # of loans sec. by farmland - orig. amts. $100K - $250K |
| 28 | Lnreag3N | # of loans sec. by farmland - orig. amts. $250K - $500K |
| 29 | Lnag4N | # of loans to finance agricultural prod. - orig. amts. of $500K or less |
| 30 | Lnag1N | # of loans to finance agricultural prod. - orig. amts. of $100K or less |
| 31 | Lnag2N | # of loans to finance agricultural prod. - orig. amts. $100K - $250K |
| 32 | Lnag3N | # of loans to finance agricultural prod. - orig. amts. $250K - $500K |
| **Loans Restructured in Troubled Debt Restructurings** | | |
| **Definition** | **Name** | **Label** |
| 1 | rslnltot | Restructured Loans & leases |
| 2 | rslnrefm | 1-4 Family loans restructured and in compliance |
| 3 | rslnls | Non 1-4 family restructured loans & leases |
| 4 | RSCONS | Construction loans restructured and in compliance |
| 5 | RSMULT | Multifamily loans restructured and in compliance |
| 6 | RSNRES | Nonfarm nonresidential loans restructured and in compliance |
| 7 | RSCI | C&I loans restructured and in compliance |
| 8 | RSOTHER | All Other loans restructured and in compliance |
| 9 | P3RSLNLT | Total restructured loans 30-89 Day P/D |
| 10 | P3RSLNFM | 1-4 Family restructured loans 30-89 Day P/D |
| 11 | p3rslnls | Restructured loans and leases, past due 30 - 89 days |
| 12 | P3RSCONS | Construction restructured loans 30-89 P/D |
| 13 | P3RSMULT | Multifamily restructured loans 30-89 Days P/D |
| 14 | P3RSNRES | Nonfarm nonresidential loans restructured loans 30-89 Day P/ |
| 15 | P3RSCI | C&I Loans restructured loans 30-89 Days P/D |
| 16 | P3RSOTH | All Other Loans restructured and Past Due 30-89days |
| 17 | P9RSLNLT | Total restructured loans 90+ days P/D |
| 18 | P9RSLNFM | Restructured Loans- 1-4 Family 90+ Days P/D |
| 19 | p9rslnls | Restructured loans and leases, past due 90+ days |
| 20 | P9RSCONS | Restructured Construction loans 90+ days P/D |
| 21 | P9RSMULT | Restructured Multifamily 90+ Days P/D |
| 22 | P9RSNRES | Restructured Nonfarm Nonresidential Loans 90+ Days P/D |
| 23 | P9RSCI | Restructured C&I Loans 90+ Days P/D |
| 24 | P9RSOTH | Restructured All Other Loans 90+ Days P/D |
| 25 | NARSLNLT | Total Nonaccrual Restructured Loans |
| 26 | NARSLNFM | Nonaccrual Restructured 1-4 Family Loans |
| 27 | narslnls | Restructured loans and leases in nonaccrual status |
| 28 | NARSCONS | Nonaccrual Restructured Construction Loans |
| 29 | NARSMULT | Nonaccrual Restructured Multifamily Loans |
| 30 | NARSNRES | Nonaccrual Restructured Non farm nonresidential Loans |
| 31 | NARSCI | Nonaccrual Restructured C&I Loans |
| 32 | NARSOTh | Restructured Nonaccrual All Other Loans |
| **Other Real Estate Owned** | | |
| **Definition** | **Name** | **Label** |
| 1 | ore | Other real estate owned |
| 2 | oreinv | Direct and indirect investments in real estate |
| 3 | oreoth | OREO: Real estate acquired |
| 4 | oreres | Other real estate owned: 1-4 family residential |
| 5 | oremult | Other Real Estate Owned: Multi-family residential |
| 6 | orenres | Other real estate owned: Commercial real estate |
| 7 | orecons | Other real estate owned:Construction & development |
| 8 | oreag | Other real estate owned: Farmland |
| 9 | oregnma | Foreclosed properties from GNMA loans before June 2018 |
| 10 | oreothf | Other real estate owned in foreign offices |
| **Goodwill and Other Intangibles** | | |
| **Definition** | **Name** | **Label** |
| 1 | intan | Goodwill and other intangibles |
| 2 | intangw | Goodwill |
| 3 | intanmsr | Mortgage servicing assets |
| 4 | intanoth | All other intangible assets |
| 5 | intangcc | Purchased credit card relationships and nonmortgage servicing before June 2018 |
| **Total Deposits** | | |
| **Definition** | **Name** | **Label** |
| 1 | dep | Total deposits |
| 2 | depdom | Deposits held in domestic offices |
| 3 | idtrni | Individuals, partnerships, and corporations |
| 4 | idtrngov | U.S. Government |
| 5 | idtrnmu | States and political subdivisions in the U.S. |
| 6 | idtrcomb | Commercial banks and other depository institutions in U.S. |
| 7 | idtrnfc | Banks in foreign countries |
| 8 | idtrnfg | Foreign governments and official institutions |
| 9 | depfor | Deposits held in foreign offices |
| 10 | depdom | Deposits held in domestic offices |
| 11 | trn | Transaction accounts |
| 12 | ddt | Demand deposits |
| 13 | ntr | Nontransaction accounts |
| 14 | ntrsmmda | Money market deposit accounts (MMDAs) |
| 15 | ntrsoth | Other savings deposits (excluding MMDAs) |
| 16 | ntrtime | Total time deposits |
| 17 | ts | Total time and savings deposits |
| 18 | depdom | Deposits held in domestic offices |
| 19 | depnidom | Noninterest-bearing deposits |
| 20 | depidom | Interest-bearing deposits |
| 21 | coredep | Retail deposits |
| 22 | depins | Estimated insured deposits |
| 23 | irakeogh | IRAs and Keogh plan accounts |
| 24 | bro | Brokered deposits |
| 25 | broins | Fully insured brokered deposits |
| 26 | DEPLSNB | Deposits obtained via list services, yet not brokered |
| 27 | depfor | Deposits held in foreign offices |
| 28 | depnifor | Noninterest-bearing deposits |
| 29 | depifor | Interest-bearing deposits |
| **Transaction Accounts** | | |
| **Definition** | **Name** | **Label** |
| 1 | trn | Transaction accounts |
| 2 | trnipcoc | Individuals, partnerships and corporations |
| 3 | trnusgov | U.S. government |
| 4 | trnmuni | States and political subdivisions in U.S. |
| 5 | trncbo | Commercial banks and other depository institutions in the U.S. |
| 6 | trnfcfg | Foreign deposits |
| 7 | trnfc | Banks in foreign countries |
| 8 | trnfg | Foreign governments and official institutions |
| **Nontransaction Accounts** | | |
| **Definition** | **Name** | **Label** |
| 1 | ntr | Nontransaction accounts |
| 2 | ntripc | Individuals, partnerships, and corporations |
| 3 | ntrusgov | U.S. Government |
| 4 | ntrmuni | States and political subdivisions in the U.S. |
| 5 | ntrcomot | Commercial banks and other depository institutions in the U.S. |
| 6 | ntrfcfg | Foreign deposits |
| 7 | ntrfc | Banks in foreign countries |
| 8 | ntrfg | Foreign governments and official institutions |
| **Time Deposits at the $100,000 Threshold** | | |
| **Definition** | **Name** | **Label** |
| 1 | ntrcdsm | Amount ($) - time deposits less than $100,000 |
| 2 | cd3less | Time deposits of < $100,000: 3 months or less |
| 3 | cd3t12s | Time deposits of < $100,000: 3 - 12 months |
| 4 | cd1t3s | Time deposits of < $100,000: 1 - 3 years |
| 5 | cdov3s | Time deposits of < $100,000: 3 or more years |
| 6 | ntrtmlg | Amount ($) - time deposits of $100,000 or more |
| 7 | cd3les | Time deposits of $100,000 or more:3 months or less |
| 8 | cd3t12 | Time deposits of $100,000 or more: 3 to 12 months |
| 9 | cd1t3 | Time deposits of $100,000 or more: 1 to 3 years |
| 10 | cdov3 | Time deposits of $100,000 or more: 3 or more years |
| **Time Deposits at the $250,000 Threshold** | | |
| **Definition** | **Name** | **Label** |
| 1 | ntrcdsm | Amount ($) - time deposits less than $100,000 |
| 2 | NTRTMMED | Amount ($) - time deposits $100,000 to $250,000 |
| 3 | idcd3less | Time deposits of < $250,000: 3 months or less |
| 4 | idcd3t12s | Time deposits of < $250,000: 3 - 12 months |
| 5 | idcd1t3s | Time deposits of < $250,000: 1 - 3 years |
| 6 | idcdov3s | Time deposits of < $250,000: 3 or more years |
| 7 | NTRTMLGJ | Time deposits over the insurance limit of $250,000 |
| 8 | idcd3les | Time deposits of $250,000 or more:3 months or less |
| 9 | idcd3t12 | Time deposits of $250,000 or more: 3 to 12 months |
| 10 | idcd1t3 | Time deposits of $250,000 or more: 1 to 3 years |
| 11 | idcdov3 | Time deposits of $250,000 or more: 3 or more years |
| **Deposits Based on the $100,000 Reporting Threshold** | | |
| **Definition** | **Name** | **Label** |
| 1 | depsmamt | Amount ($) Deposit accounts of $100,000 or less |
| 2 | depsmb | Number (#) of deposit accounts of $ 100,000 or less (June only for Call) |
| 3 | deplgamt | Amount ($) deposit accounts of more than $100,000 |
| 4 | deplgb | Number (#) of deposit accounts more then $100,000 |
| **Deposits Based on the $250,000 Reporting Threshold** | | |
| **Definition** | **Name** | **Label** |
| 1 | iddepsam | Amount ($) - deposit accounts equal to or less than $250,000 |
| 2 | iddepsmb | Number (#) - deposit accounts equal to or less than $250,000 |
| 3 | DEPSMRA | Amount ($) of retirement deposit accounts of $250,000 or less |
| 4 | DEPSMRN | Number (#) of retirement deposit accounts of $250,000 or less |
| 5 | NTRCDSMJ | Time deposits less than or equal to insurance limit |
| 6 | iddeplam | Amount ($) - deposit accounts of more than $250,000 |
| 7 | iddeplgb | Number (#) - deposit accounts of more than $250,000 |
| 8 | DEPLGRA | Amount ($) of retirement deposit accounts of more than $250,000 |
| 9 | DEPLGRN | Number (#) of retirement deposit accounts of more than $250,000 |
| 10 | TRNNIA | Amount of noninterest-bearing transaction deposit accounts over $250,000 |
| 11 | TRNNIN | Number of noninterest-bearing transaction deposit accounts over $250,000 |
| 12 | NTRTMLGJ | Time deposits over the insurance limit of $250,000 |
| **Deposits Held in Foreign Offices** | | |
| **Definition** | **Name** | **Label** |
| 1 | depfor | Deposits held in foreign offices |
| 2 | depipccf | Individuals, partnerships and corporations |
| 3 | depusbkf | Commercial banks and other depository institutions in U.S. |
| 4 | depfbkf | Banks in foreign countries |
| 5 | depfgovf | Foreign governments and official institutions |
| 6 | depusmf | U.S government, states, and political subdivisions |
| **Changes in Bank Equity Capital** | | |
| **Definition** | **Name** | **Label** |
| 1 | eqcprev/idnull | Amended balance at previous year-end |
| 2 | eqcrest/idnull | Restatements from amended reports of income, net |
| 3 | netinc/idnull | Net income |
| 4 | eqcstkrx/idnull | Sale, conversion, retirement of capital stock, net |
| 5 | eqctrstx/idnull | Net treasury stock transactions |
| 6 | eqcmrg/idnull | Changes incidental to business combinations, net |
| 7 | eqcdivp/idnull | Cash dividends declared on preferred stock |
| 8 | eqcdivc/idnull | Cash dividends declared on common stock |
| 9 | eqccompi/idnull | Other comprehensive income |
| 10 | eqcbhctr/idnull | Other transactions with parent holding company |
| 11 | eq/idnull | Bank equity capital |
| **Total Unused Commitments** | | |
| **Definition** | **Name** | **Label** |
| 1 | uc | Total unused commitments |
| 2 | ucloc | Loan commitments-revolving, open-end lines secured by 1-4's |
| 3 | uccrcd | Credit card lines |
| 4 | uccomre | Commercial real estate, construction & land development |
| 5 | uccomres | Commitments secured by real estate |
| 6 | uccomreu | Commitments not secured by real estate |
| 7 | ucsc | Securities underwriting |
| 8 | ucother | Other unused commitments |
| 9 | ucover1 | Commitments with an original maturity exceeding 1 yr |
| 10 | sclent | Securities lent |
| 11 | othoffbs | All other off-balance sheet liabilities |
| 12 | partconv | Conveyed to others by reporting bank |
| 13 | partacqu | Acquired by reporting bank |
| **Letters of Credit** | | |
| **Definition** | **Name** | **Label** |
| 1 | locfpsb | Financial and performance standby letters of credit & foreign office guarantees |
| 2 | locfpsbk | Amount conveyed to others |
| 3 | locfsb | Financial standby letters of credit & foreign office guarantees |
| 4 | locfsbk | Amount conveyed to others |
| 5 | locpsb | Performance standby letters of credit |
| 6 | locpsbk | Amount conveyed to others |
| 7 | loccom | Commercial and similar letters of credit |
| **Total Assets and Liabilities in Foreign Offices** | | |
| **Definition** | **Name** | **Label** |
| 1 | assetfor | Total assets in foreign offices |
| 2 | chbalfor | Cash and due from depository institutions |
| 3 | repopurf | Reverse repo agreements purchased - foreign offices |
| 4 | lnlsgrf | Total loans and leases |
| 5 | unincfor | Unearned income |
| 6 | lnlsfor | Loans and leases, gross |
| 7 | lnrefor | Loans held in foreign offices |
| 8 | idlndacf | Loans to Depository Institutions & Acceptances of Other Banks � Foreign offices |
| 9 | lndepcbf | To commercial banks in U.S. |
| 10 | lndepusf | To other depository institutions in U.S. |
| 11 | lndepfcf | To banks in foreign countries |
| 12 | lnagfor | Farm loans |
| 13 | lncifor | Commercial and industrial loans |
| 14 | lncinusf | To non-U.S. addressees |
| 15 | lnconfor | Loans to individuals |
| 16 | lnfgfor | Loans to foreign governments and official institutions |
| 17 | lnmunif | Obligations of states and political subdivisions in U.S. |
| 18 | lnotherf | Other loans |
| 19 | lsfor | Lease financing receivables |
| 20 | trfor | Trading account assets |
| 21 | liabfor | Total liabilities in foreign offices |
| 22 | depfor | Deposits held in foreign offices |
| 23 | depipccf | Individuals, partnerships and corporations |
| 24 | depusbkf | Commercial banks and other depository institutions in U.S. |
| 25 | depfbkf | Banks in foreign countries |
| 26 | depfgovf | Foreign governments and official institutions |
| 27 | depusmf | U.S government, states, and political subdivisions |
| 28 | depfor | Deposits held in foreign offices |
| 29 | depnifor | Noninterest-bearing deposits |
| 30 | depifor | Interest-bearing deposits |
| 31 | reposldf | Reverse repo agreements sold - foreign offices |
| 32 | othborf | Other borrowed funds |
| **Derivatives** | | |
| **Definition** | **Name** | **Label** |
| 1 | obsdir | Derivatives |
| 2 | idctder | Notional amount of credit derivatives |
| 3 | ctdergty | Bank is guarantor |
| 4 | ctderben | Bank is beneficiary |
| 5 | rt | Interest rate contracts |
| 6 | rtnvs | Notional value of interest rate swaps |
| 7 | rtffc | Futures and forward contracts |
| 8 | rtwoc | Written option contracts |
| 9 | rtpoc | Purchased option contracts |
| 10 | fx | Foreign exchange rate contracts |
| 11 | fxnvs | Notional value of exchange swaps |
| 12 | fxffc | Commitments to purchase foreign currencies & U.S. dollar exchange |
| 13 | fxspot | Spot foreign exchange rate contracts |
| 14 | fxwoc | Written option contracts |
| 15 | fxpoc | Purchased option contracts |
| 16 | edcm | Contracts on other commodities and equities. |
| 17 | othnvs | Notional value of other swaps |
| 18 | othffc | Futures and forward contracts |
| 19 | othwoc | Written option contracts |
| 20 | othpoc | Purchased option contracts |
| **Past Due and Nonaccrual Assets** | | |
| **Definition** | **Name** | **Label** |
| 1 | p3asset | Assets past due 30-89 days |
| 2 | p3re | Loans secured by real estate, total past due 30 - 89 days |
| 3 | p3renus | To non-U.S. addressees, past due 30 - 89 days |
| 4 | idp3redm | Real estate loans in domestic offices, past due 30 - 89 days |
| 5 | p3recons | Construction and land development, past due 30 - 89 days |
| 6 | P3RECNFM | 1-4 family residential construction loans past due 30 through 89 days |
| 7 | P3RECNOT | Other construction all land development and other land loans past due 30-89 days |
| 8 | p3reag | Secured by farmland, past due 30 - 89 days |
| 9 | p3reres | Secured by 1-4 family residential properties, past due 30 - 89 days |
| 10 | p3remult | Secured by multifamily residential properties, past due 30 - 89 days |
| 11 | p3renres | Secured by nonfarm nonresidential properties, past due 30 - 89 days |
| 12 | P3RENROW | Secured by owner-occupied nonfarm nonresidential properties, past due 30-89 days |
| 13 | P3RENROT | Secured by other nonfarm nonresidential properties, past due 30-89 days |
| 14 | p3refor | Real estate loans in foreign offices, past due 30 - 89 days |
| 15 | p3dep | Loans to depository institutions, past due 30 - 89 days |
| 16 | p3depnus | Loans to foreign banks, past due 30 - 89 days |
| 17 | p3ag | Loans to finance agricultural production and other loans to farmers, past due 30 |
| 18 | p3ci | Commercial and industrial loans, past due 30 - 89 days |
| 19 | p3cinus | To non-U.S. addressees, past due 30 - 89 days |
| 20 | p3con | Loans to individuals, past due 30 - 89 days |
| 21 | p3crcd | Credit cards and related plans, past due 30 - 89 days |
| 22 | P3AUTO | 30-89 Days P/D Auto Loans |
| 23 | P3CONOTH | 30-89 Days P/D-Other Consumer |
| 24 | p3fg | Loans to foreign governments and official institutions, past due 30 - 89 days |
| 25 | p3othln | Other loans, past due 30 - 89 days |
| 26 | p3ls | Lease financing receivables, past due 30 - 89 days |
| 27 | p3scdebt | Debt securities and other assets, past due 30 - 89 days |
| 28 | p3agsm | Loans for agricultural production and other loans to farmers, past due 30-89days |
| 29 | p3lnsale | Loans held for sale past due 30-89 days |
| 30 | P3GTYPAR | Loans and leases 30-89 past due, U.S. Government wholly or partially guaranteed |
| 31 | P3LTOT | 30-89 D P/D Total Loans- FDIC Loss Sharing |
| 32 | p9asset | Assets past due 90 or more days |
| 33 | p9re | Loans secured by real estate, total past due 90+ days |
| 34 | p9renus | Loans to non-U.S. addressees, past due 90+ days |
| 35 | idp9redm | Real estate loans in domestic offices, past due 90+ days |
| 36 | p9recons | Construction and land development, past due 90+ days |
| 37 | P9RECNFM | 1-4 family residential construction loans past due 90 days or more |
| 38 | P9RECNOT | Other construction, all land development and other land loans past due 90+ days |
| 39 | p9reag | Loans secured by farmland, past due 90+ days |
| 40 | p9reres | Secured by 1-4 family residential properties, past due 90+ days |
| 41 | p9remult | Secured by multifamily residential properties, past due 90+ days |
| 42 | p9renres | Loans secured by nonfarm nonresidential properties, past due 90+ days |
| 43 | P9RENROW | Secured by owner-occupied nonfarm nonresidential properties, past due 90+ days |
| 44 | P9RENROT | Secured by other nonfarm nonresidential properties, past due 90+ days |
| 45 | p9refor | Real estate loans in foreign offices, past due 90+ days |
| 46 | p9dep | Loans to depository institutions, past due 90+ days |
| 47 | p9depnus | Loans to foreign banks, past due 90+ days |
| 48 | p9ag | Loans to finance agricultural and other loans to farmers, past due 90+ days |
| 49 | p9ci | Commercial and industrial loans, past due 90+ days |
| 50 | p9cinus | To non-U.S. addressees, past due 90+ days |
| 51 | p9con | Loans to individuals, past due 90+ days |
| 52 | p9crcd | Credit cards and related plans, past due 90+ days |
| 53 | P9AUTO | 90+ Days P/D Auto Loans |
| 54 | P9CONOTH | 90+ Days P/D-Other Consumer |
| 55 | p9fg | Loans to foreign governments and official institutions, past due 90+ days |
| 56 | p9othln | Other loans, past due 90+ days |
| 57 | p9ls | Lease financing receivables, past due 90+ days |
| 58 | p9scdebt | Debt securities and other assets, past due 90+ days |
| 59 | p9agsm | Loans for agriculture and other loans to farmers, past due 90+ days |
| 60 | p9lnsale | Loans held for sale, past due 90 or more days |
| 61 | P9GTYPAR | Loans and leases, wholly or partially guaranteed by the U.S. Government-90+PD |
| 62 | P9LTOT | 90+ D P/D Total Loans � FDIC Loss Sharing |
| 63 | naasset | Assets in nonaccrual status |
| 64 | nare | Loans secured by real estate, total in nonaccrual status |
| 65 | narenus | Laons to non-U.S. addressees, in nonaccrual status |
| 66 | idnaredm | Real estate loans in domestic offices in nonaccrual status |
| 67 | narecons | Construction and land development in nonaccrual status |
| 68 | NARECNFM | Nonaccrual 1-4 family residential construction loans |
| 69 | NARECNOT | Nonaccrual other construction, all land development and other land loans |
| 70 | nareag | Loans secured by farmland in nonaccrual status |
| 71 | nareres | Secured by 1-4 family residential properties in nonaccrual status |
| 72 | naremult | Secured by multifamily residential properties in nonaccrual status |
| 73 | narenres | Loans secured by nonfarm nonresidential properties in nonaccrual status |
| 74 | NARENROW | Loans secured by owner-occupied nonfarm nonresidential properties, in nonaccrual |
| 75 | NARENROT | Loans secured by other nonfarm nonresidential properties, in nonaccrual status |
| 76 | narefor | Real estate loans in foreign offices in nonaccrual status |
| 77 | nadep | Loans to depository institutions in nonaccrual status |
| 78 | nadepnus | Loans to foreign banks in nonaccrual status |
| 79 | naag | Loans for agriculture and other loans to farmers in nonaccrual status |
| 80 | naci | Commercial and industrial loans in nonaccrual status |
| 81 | nacinus | Loans to non-U.S. addressees in nonaccrual status |
| 82 | nacon | Loans to individuals in nonaccrual status |
| 83 | nacrcd | Credit cards and related plans in nonaccrual status |
| 84 | NAAUTO | Nonaccrual Auto Loans |
| 85 | NACONOTH | Nonaccrual-Other Consumer |
| 86 | nafg | Loans to foreign governments and official institutions in nonaccrual status |
| 87 | naothln | Other loans in nonaccrual status |
| 88 | nals | Lease financing receivables in nonaccrual status |
| 89 | nascdebt | Debt securities and other assets in nonaccrual status |
| 90 | naagsm | Loans to finance agriculture and other loans to farmers in nonaccrual status |
| 91 | nalnsale | Nonaccrual Loans held for sale in nonaccrual status |
| 92 | NAGTYPAR | Guaranteed portion of nonaccrual loans and leases, excluding GNMA loans |
| 93 | NALTOT | Total Nonaccrual Loans � FDIC Loss Sharing |
| **Past Due 30-89 Days 1-4 Family Residential** | | |
| **Definition** | **Name** | **Label** |
| 1 | p3reres | Secured by 1-4 family residential properties, past due 30 - 89 days |
| 2 | p3rersfm | Past due 30-89 days secured by 1-4 Family first liens |
| 3 | p3rersf2 | Past due 30-89 days secured by 1-4 Family junior liens |
| 4 | p3reloc | Past due 30-89 days home equity lines of credit |
| **Past Due 90+ Days 1-4 Family Residential** | | |
| **Definition** | **Name** | **Label** |
| 1 | p9reres | Secured by 1-4 family residential properties, past due 90+ days |
| 2 | p9rersfm | Past due 90+ days secured by 1-4 Family first liens |
| 3 | p9rersf2 | Past due 90+ days secured by 1-4 Family junior liens |
| 4 | p9reloc | Past due 90+ days home equity lines of credit |
| **Nonaccrual 1-4 Family Residential** | | |
| **Definition** | **Name** | **Label** |
| 1 | nareres | Secured by 1-4 family residential properties in nonaccrual status |
| 2 | narersfm | Nonaccrual loans secured by 1-4 Family first liens |
| 3 | narersf2 | Nonaccrual loans secured by 1-4 Family junior liens |
| 4 | nareloc | Nonaccrual home equity lines of credit |
| **- PD & NA Loans Wholly or Partially US Gvmt Guaranteed** | | |
| **Definition** | **Name** | **Label** |
| 1 | P3GTYPAR | Loans and leases 30-89 past due, U.S. Government wholly or partially guaranteed |
| 2 | p3gty | Guaranteed portion of loans and leases, excluding GNMA loans, 30-89days past due |
| 3 | P3GTYGNM | Rebooked GNMA loans repurchased or are eligible for repurchase, 30-89 days pas |
| 4 | P9GTYPAR | Loans and leases, wholly or partially guaranteed by the U.S. Government-90+PD |
| 5 | p9gty | Guaranteed portion of loans and leases, excluding GNMA loans, 90 days past due |
| 6 | P9GTYGNM | Rebooked GNMA loans that have been repurchased or are eligible for repurchase, |
| 7 | NAGTYPAR | Guaranteed portion of nonaccrual loans and leases, excluding GNMA loans |
| 8 | nagty | Nonaccrual Loans and leases, wholly or partially guaranteed by the US Government |
| 9 | NAGTYGNM | Nonaccrual rebooked GNMA loans |
| **Fiduciary and Related Services** | | |
| **Definition** | **Name** | **Label** |
| 1 | trpower | Fiduciary powers granted |
| 2 | trexer | Fiduciary power exercised |
| 3 | tract | Fiduciary or related activity |
| 4 | idfranum | Number of Fiduciary Accounts and Related Asset Accounts |
| 5 | ttnanum | Number of Managed Accounts |
| 6 | ttnmnum | Number of Non-Managed Accounts |
| 7 | idtfra | Total fiduciary and related assets |
| 8 | ttma | Managed Assets ($) |
| 9 | ttnma | Non-managed Assets ($) |
| 10 | ifiduc | Gross Fiduciary activities income |
| **Number of Fiduciary and Related Asset Accounts** | | |
| **Definition** | **Name** | **Label** |
| 1 | trpower | Fiduciary powers granted |
| 2 | trexer | Fiduciary power exercised |
| 3 | tract | Fiduciary or related activity |
| 4 | idfranum | Number of Fiduciary Accounts and Related Asset Accounts |
| 5 | ttnanum | Number of Managed Accounts |
| 6 | tpmanum | Personal Trust and Agency Accounts |
| 7 | tecmanum | Employee Benefit-defined Contribution Accounts |
| 8 | tebmanum | Employee Benefit-defined Benefit Accounts |
| 9 | tormanum | Number of other employee benefit and retirement-related accounts |
| 10 | tcamanum | Corporate Trust and Agency Accounts |
| 11 | timmanum | Number of managed investment management and investment advisory agency accounts |
| 12 | TFEMANUM | # of foundation and endowment trust and agency accounts, managed assets |
| 13 | tofmanum | Other Fiduciary Accounts |
| 14 | tmafnum | Fiduciary Accounts Held in Foreign Offices |
| 15 | TRHMANUM | # of Individual retirement, health savings, and similar accounts,managed assets |
| 16 | TMASMFN | # of investments of managed fiduciary accts in advised or sponsored mutual funds |
| 17 | ttnmnum | Number of Non-Managed Accounts |
| 18 | tpnmnum | Personal Trust and Agency Accounts |
| 19 | tecnmnum | Employee Benefit-defined Contribution Accounts |
| 20 | tebnmnum | Employee Benefit-defined Benefit Accounts |
| 21 | tornmnum | Number non-managed other employee benefit and retirement-related accounts |
| 22 | tcanmnum | Corporate Trust and Agency Accounts |
| 23 | TIMNMNUM | # of investment management and advisory agency accounts, non-managed assets |
| 24 | TFENMNUM | Number (#) individual retirement, health savings, and other similar accounts |
| 25 | tofnmnum | Other Fiduciary Accounts |
| 26 | tcsnmnum | Custody and Safekeeping Accounts |
| 27 | tnmnumf | Fiduciary Accounts Held in Foreign Offices |
| 28 | TRHNMNUM | # of Individual retirement,health savings and similar accounts, non-managed asts |
| **Total Fiduciary and Related Assets** | | |
| **Definition** | **Name** | **Label** |
| 1 | trpower | Fiduciary powers granted |
| 2 | trexer | Fiduciary power exercised |
| 3 | tract | Fiduciary or related activity |
| 4 | idtfra | Total fiduciary and related assets |
| 5 | ttma | Managed Assets ($) |
| 6 | tpma | Personal trust and agency accounts, managed assets |
| 7 | tecma | Employee benefit-defined contribution accounts, managed assets |
| 8 | tebma | Employee benefit-defined benefit accounts, managed assets |
| 9 | torma | Other employee benefit and retirement-related accounts, managed assets |
| 10 | tcama | Corporate trust and agency accounts, managed assets |
| 11 | timma | Investment management and investment advisory agency accounts, managed assets |
| 12 | TFEMA | Foundation and endowment trust and agency accounts, managed assets |
| 13 | tofma | Other fiduciary accounts, managed assets |
| 14 | tmaf | Fiduciary accounts held in foreign offices, managed assets |
| 15 | TRHMA | Individual retirement,health savings and similar accounts, managed assets |
| 16 | TMASMF | Investments of managed fiduciary accts in advised or sponsored mutual funds |
| 17 | ttnma | Non-managed Assets ($) |
| 18 | tpnma | Personal trust and agency accounts, non-managed assets |
| 19 | tecnma | Employee benefit-defined contribution accounts, non-managed assets |
| 20 | tebnma | Employee benefit-defined benefit accounts, non-managed Assets |
| 21 | tornma | Other retirement accounts, non-managed assets |
| 22 | tcanma | Corporate trust and agency accounts, non-managed assets |
| 23 | TIMNMA | Investment management and advisory agency accounts, non-managed assets |
| 24 | TFENMA | Foundation and endowment trust and agency accounts, non-managed assets |
| 25 | tofnma | Other fiduciary accounts, non-managed assets |
| 26 | tcsnma | Custody and safekeeping accounts, non-managed assets |
| 27 | tnmaf | Fiduciary accounts held in foreign offices, non-managed Assets |
| 28 | TRHNMA | Individual retirement,health savings and similar accounts, non-managed |
| **Total Managed Assets held in Fiduciary Accounts** | | |
| **Definition** | **Name** | **Label** |
| 1 | trpower | Fiduciary powers granted |
| 2 | trexer | Fiduciary power exercised |
| 3 | tract | Fiduciary or related activity |
| 4 | tpimatot | Total managed assets held in fiduciary accounts |
| 5 | TPINI | Managed personal trust&agency accnts & investmnt managemnt agency accounts |
| 6 | tpii | Interest-bearing deposits managed personal trust and agency accounts and investm |
| 7 | TPISCUS | US Treasury & US government agency obligations, managed personal trust&agency |
| 8 | TPISCMUN | State, county and municipal obligations, managed personal trust&agency accts, |
| 9 | TPIMMF | Money market mutual funds, managed personal trust&agency accts, etc.. |
| 10 | TPIEQF | Equity mutual funds, managed personal trust&agency accts, etc.. |
| 11 | TPIOTHF | Other mutual funds, managed personal trust&agency accts, etc.. |
| 12 | TPITRF | Common trust funds and collective investment funds, managed personal trust&ag |
| 13 | TPISTO | Other Short-term obligations, managed personal trust&agency accts, etc.. |
| 14 | TPIOTHB | Other Notes and Bonds, managed personal trust&agency accts, etc.. |
| 15 | TPIUF | Investmnts in unregistered funds&private equity investmnts,managd personal trust |
| 16 | TPICPS | Other common and preferred stocks, managed personal trust&agency accts, etc.. |
| 17 | TPIREMTG | Real Estate Mortgages, managed personal trust&agency accts, etc.. |
| 18 | TPIRE | Real Estate, managed personal trust&agency accts, etc.. |
| 19 | TPIMISC | Miscellaneous assets, managed personal trust&agency accts, etc.. |
| 20 | TEMATOT | Managed employee benefit and retirements-related trust and agency acccounts |
| 21 | TENI | Managed employee benefit & retirement trust noninterest bearing deposits |
| 22 | tei | Employee Benefit & Retirement Trust Interest Bearing Deposits |
| 23 | tescus | U.S. treasury and U.S. government agency obligations,managed emp benft&retire |
| 24 | TESCMUN | State, county & municipal obligations,managed emp benft & retire-related |
| 25 | TEMMF | Money market mutual Funds,managed emp benft & retire-related trust accnts |
| 26 | TEEQF | Equity mutual funds,managed emp benft & retire-related trust accnts |
| 27 | TEOTHF | Other mutual funds,managed emp benft & retire-related trust accnts |
| 28 | TETRF | Common trust funds & collective investment funds,managd emp benft & retiremnt |
| 29 | TESTO | Other short-term obligations, emp benft & retire-related trust accnts |
| 30 | TEOTHB | Other notes and bonds,managed emp benft & retire-related trust accnts |
| 31 | TEUF | Investments in unregistered funds & private equity investmnts,emp benft & retire |
| 32 | TECPS | Other common & preferred stocks,managed emp benft & retire-related trust accts |
| 33 | TEREMTG | Real estate mortgages,managed emp benft & retire-related trust accnts |
| 34 | TERE | Real Estate,managed emp benft & retire-related trust accnts |
| 35 | TEMISC | Miscellaneous assets,managed emp benft & retire-related trust accnts |
| 36 | TOMATOT | Total all other managed assets: |
| 37 | TONI | Noninterest-bearing Deposits, all other managed accounts |
| 38 | TOI | Interest-bearing deposits, all other managed accounts |
| 39 | TOSCUS | U.S. treasury & U.S. government agency obligations, all other managed accounts |
| 40 | TOSCMUN | State, county and municipal obligations, all other managed accounts |
| 41 | TOMMF | Money market mutual funds, all other managed accounts |
| 42 | TOEQF | Equity mutual funds, all other managed accounts |
| 43 | TOOTHF | Other mutual funds, all other managed accounts |
| 44 | TOTRF | Common trust funds and collective investment funds, all other managed account |
| 45 | TOSTO | Other short-term obligations, all other managed accounts |
| 46 | TOOTHB | Other notes and bonds, all other managed accounts |
| 47 | TOUF | Investmnts in unregistered funds&private equity investmnts, all other managed |
| 48 | TOCPS | Other common and preferred stocks, all other managed accounts |
| 49 | TOREMTG | Real Estate Mortgages, all other managed accounts |
| 50 | TORE | Real estate, all other managed accounts |
| 51 | TOMISC | Miscellaneous assets, all other managed accounts |
| **Corporate Trust and Agency Accounts** | | |
| **Definition** | **Name** | **Label** |
| 1 | trpower | Fiduciary powers granted |
| 2 | trexer | Fiduciary power exercised |
| 3 | tract | Fiduciary or related activity |
| 4 | tcapao | Principal Amount Outstanding |
| 5 | TCAPAOD | Amount of corporate trust and agency account issues in default |
| 6 | tcanum | Corporate and Municipal Trusteeships |
| 7 | TCANUMD | Number of corporate trust and agency account issues in default |
| 8 | tcatnum | Transfer Agent, Registrar, Paying Agent and Other Corporate Agency |
| **Collective Investment & Common Trust Funds** | | |
| **Definition** | **Name** | **Label** |
| 1 | trpower | Fiduciary powers granted |
| 2 | trexer | Fiduciary power exercised |
| 3 | tract | Fiduciary or related activity |
| 4 | tctotnum | Total Number of Collective Investment Funds and Common Trust Funds |
| 5 | tcdenum | Domestic Equity |
| 6 | tcienum | International/Global Equity |
| 7 | tcsbnum | Stock/Bond Blend |
| 8 | tctbnum | Taxable Bond |
| 9 | tcmbnum | Municipal Bond |
| 10 | tcstnum | Short-term Investments/Money Market |
| 11 | tcsonum | Specialty/Other |
| 12 | tctotmv | Total Market Value of Collective Investment Fund and Common Trust Funds |
| 13 | tcdemv | Domestic Equality |
| 14 | tciemv | International/Global Equity |
| 15 | tcsbmv | Stock/Bond Blend |
| 16 | tctbmv | Taxable Bond |
| 17 | tcmbmv | Municipal Bond |
| 18 | tcstmv | Short-term Investments/Money Market |
| 19 | tcsomv | Specialty/Other |
| **Gross Fiduciary and Related Services Income** | | |
| **Definition** | **Name** | **Label** |
| 1 | trpower | Fiduciary powers granted |
| 2 | trexer | Fiduciary power exercised |
| 3 | tract | Fiduciary or related activity |
| 4 | tip | YTD Gross Income from Personal trust and agency accounts |
| 5 | tiec | YTD Gross Income employee benefit defined contribution |
| 6 | tieb | YTD Gross Income employee benefit defined benefit |
| 7 | tior | YTD Gross Income from other retirement accounts |
| 8 | tica | YTD Gross Income corporate trust and agency |
| 9 | TIMA | Investment management and investment advisory agency accounts income |
| 10 | TIFE | Foundation and endowment and agency accounts income |
| 11 | tiof | YTD Gross income other trust and agency accounts |
| 12 | tics | YTD Gross income custody and safekeeping agency accounts |
| 13 | tir | YTD Gross income other trust accounts |
| 14 | ifiduc | Gross Fiduciary activities income |
| 15 | tetot | YTD fiduciary expense (Dec. only) |
| 16 | tnl | YTD fiduciary net loss (Dec. only) |
| 17 | tintra | YTD fiduciary intracompany income (Dec. only) |
| 18 | tni | YTD net fiduciary income (Dec. only) |
| 19 | titotf | YTD foreign offices total gross fiduciary income |
| **Fiduciary settlements, surcharges, and other losses** | | |
| **Definition** | **Name** | **Label** |
| 1 | trpower | Fiduciary powers granted |
| 2 | trexer | Fiduciary power exercised |
| 3 | tract | Fiduciary or related activity |
| 4 | TTOTMAGL | Gross losses for managed accounts |
| 5 | TPMAGL | Fiduciary settlements, surcharges, and other losses, managed accounts |
| 6 | TRTMAGL | Employee benefit and retirement-related gross losses for managed accounts |
| 7 | TIMMAGL | Gross losses for investment management and investment managed accounts |
| 8 | TOFMAGL | Gross losses for other fiduciary accounts and related managed accounts |
| 9 | TTOTNMGL | Gross losses for non-managed accounts |
| 10 | TPNMGL | Fiduciary settlements, surcharges, and other losses |
| 11 | trtnmgl | Employee benefit and retirement-related trust and agency accounts, non-managed |
| 12 | TIMNMGL | Investmnt managmnt & investmnt advisory agency accts-gross losses nonmanaged a |
| 13 | TOFNMAGL | Other fiduciary accounts and related services-gross losses nonmanaged asts |
| 14 | TTOTREC | Recoveries fiduciary and related services income |
| 15 | TPTREC | Fiduciary settlements, surcharges, and other - recoveries |
| 16 | TRTREC | Employee benefit and retirement-related trust and agency accounts-recoveries |
| 17 | TIMREC | Investment management and investment advisory agency accounts recoveries |
| 18 | TOFREC | Other fiduciary accounts and related services-recoveries |
| **Covered by FDIC Loss-Share Agreements** | | |
| **Definition** | **Name** | **Label** |
| 1 | LSALNLS | Carry amount for loans and leases covered by loss share agreements |
| 2 | LRECONS | Re construction Loan - FDIC loss-sharing agreements |
| 3 | LREAG | Re Farmland Loans - FDIC loss-sharing agreements |
| 4 | LRERES | RE 1-4 Family Loans - FDIC loss-sharing agreements |
| 5 | LREMULT | RE Multifamily Loans-FDIC loss-sharing agreements |
| 6 | LRENRES | RE Nonfarm Nonresidential Loans - FDIC loss-sharing agreements |
| 7 | LCI | C&I Loans - FDIC loss-sharing agreements |
| 8 | LCON | Consumer Loans - FDIC loss-sharing agreements |
| 9 | lag | Covered amount of Farm Loans under FDIC loss sharing agreement |
| 10 | LOTH | All Other Ln & Ls - FDIC loss-sharing agreements |
| 11 | LSAORE | Carry amount for ORE covered by loss share agreements |
| 12 | LOREGTY | ORE Protected amount - FDIC loss-sharing agreements |
| 13 | LSASCDBT | Carry amount for dept securities covered by loss share agreements |
| 14 | LSAOA | Carry amount for other assets covered by loss share agreements |
| 15 | P3LTOT | 30-89 D P/D Total Loans- FDIC Loss Sharing |
| 16 | P3LRECON | 30-89 P/D Construction -FDIC loss-sharing agreements |
| 17 | P3LREAG | 30-89 Day P/D RE Farm loans-FDIC loss-sharing agreements |
| 18 | p3lreres | 30-89 P/D 1-4 Family residential loans - FDIC loss-sharing agreements |
| 19 | P3LREMUL | 30-89 Day P/D Multifamily loans - FDIC loss-sharing agreements |
| 20 | P3LRENRS | 30-89 P/D Nonfarm Nonresidential loans - FDIC loss-sharing agreements |
| 21 | P3LCI | 30-89 Days P/D C&I Loans- FDIC loss-sharing agreements |
| 22 | P3LCON | 30-89 D P/D Consumer loans - FDIC loss-sharing agreements |
| 23 | p3lag | 30-89 Days PD Farm loans - FDIC loss-sharing agreements |
| 24 | P3LOTH | 30-89 D P/D Other Loans - FDIC loss-sharing agreements |
| 25 | P3LGTY | 30-89 P/D protected portion (GTY)- FDIC Loss Sharing Agreements |
| 26 | P9LTOT | 90+ D P/D Total Loans � FDIC Loss Sharing |
| 27 | p9lrecon | 90+ Days PD Construction loans-FDIC loss sharing agreements |
| 28 | P9LREAG | 90+ D P/D RE Farm Loans - FDIC Loss Sharing Agreements |
| 29 | P9LRERES | 90+ D P/D 1-4 Family Loans � FDIC Loss Sharing Agreements |
| 30 | P9LREMUL | 90+ D P/D Multifamily Loans � FDIC Loss Sharing Agreements |
| 31 | P9LRENRS | 90+ D P/D Nonfarm Nonresidential Properties � FDIC Loss Sharing Agreements |
| 32 | P9LCI | 90+ D P/D C&I - FDIC Loan Loss Sharing Agreement |
| 33 | P9LCON | 90+ D P/D Consumer Loans � FDIC Loss Sharing Agreements |
| 34 | p9lag | 90+ Days P/D Farm loans - FDIC loss-sharing agreements |
| 35 | P9LOTH | 90+ D P/D Other Loans and Leases � FDIC Loss Sharing Agreements |
| 36 | P9LGTY | 90+ D P/D protected portion of covered loans (GTYY)-FDIC Loss Sharing Agreements |
| 37 | NALTOT | Total Nonaccrual Loans � FDIC Loss Sharing |
| 38 | NALRECON | Nonaccrual Construction Land Development Loss-Sharing Agreements |
| 39 | NALREAG | Nonaccrual RE Farm Loans - FDIC Loss Sharing Agreements |
| 40 | NALRERES | Nonaccrual 1-4 Family Loans � FDIC Loss Sharing Agreement |
| 41 | NALREMUL | Nonaccrual Multifamily Loans � FDIC Loss Sharing Agreements |
| 42 | NALRENRS | Nonfarm Nonresidential Properties Loans � FDIC Loss Sharing Agreements |
| 43 | NALCI | Nonaccrual C&I Loans � FDIC Loss Sharing Agreements |
| 44 | NALCON | Nonaccrual Consumer Loans - FDIC Loss Sharing Agreements |
| 45 | nalag | Nonaccrual Farm loans - FDIC loss-sharing agreements |
| 46 | NALOTH | Nonaccrual Other Loans � FDIC Loss Sharing Agreements |
| 47 | NALGTY | Nonaccrual protected portion (GTY)- FDIC loss sharing agreements |
| **Bank Assets Sold and Securitized** | | |
| **Definition** | **Name** | **Label** |
| 1 | szlnres | Bank Securitization Activities, Princiapl Balance for 1-4 Family Res. |
| 2 | szlnhel | Bank Securitization Activities, Principal Balance for Home Equity Lines |
| 3 | szlncrcd | Bank Securitization Activities, Principal Balance for Credit Cards Rec. |
| 4 | szlauto | Bank Securitization Activities , Principal Balance Auto Loans |
| 5 | szlncon | Bank Securitization Activities , Principal Balance Other Consumer Loans |
| 6 | szlnci | Bank Securitization Activities , Principal Balance C&I Loans |
| 7 | szlnoth | Bank Securitization Activities , Principal Balance All Other Lns & Leases |
| 8 | sz30res | Past Due Loan Amounts 30-89 Days 1-4 Family Residential Loans |
| 9 | sz30hel | Past Due Loan Amounts 30-89 Days Home Equity Lines |
| 10 | sz30crcd | Past Due Loan Amounts 30-89 Days Credit Card Receivables |
| 11 | sz30auto | Past Due Loan Amounts 30-89 Days Auto Loans |
| 12 | sz30con | Past Due Loan Amounts 30-89 Days Other Consumer Loans |
| 13 | sz30ci | Past Due Loan Amounts 30-89 Days C&I Loans |
| 14 | sz30oth | Past Due Loan Amounts 30-89 Days All Other Loans and All Leases |
| 15 | sz90res | Past Due Loan Amounts 90 Days or More |
| 16 | sz90hel | Past Due Loan Amounts 90 Days or More Home Equity Lines |
| 17 | sz90crcd | Past Due Loan Amounts 90 Days or More Credit Cards Receivables |
| 18 | sz90auto | Past Due Loan Amounts 90 Days or More Auto Loans |
| 19 | sz90con | Past Due Loan Amounts 90 Days or More Other Consumer Loans |
| 20 | sz90ci | Past Due Loan Amounts 90 Days or More C&I Loans |
| 21 | sz90oth | Past Due Loan Amounts 90 Days or More All Other Loans and All Leases |
| 22 | szdrres | Charge-Offs On Assets Sold And Securitized 1-4 Family Residential Loans |
| 23 | szdrhel | Charge-Offs On Assets Sold And Securitized Home Equity Lines |
| 24 | szdrcrcd | Charge-Offs On Assets Sold And Securitized Credit Cards Receivables |
| 25 | szdrauto | Charge-Offs On Assets Sold And Securitized Auto Loans |
| 26 | szdrcon | Charge-Offs On Assets Sold And Securitized Other Consumer Loans |
| 27 | szdrci | Charge-Offs On Assets Sold And Securitized C&I Loans |
| 28 | szdroth | Charge-Offs On Assets Sold And Securitized All Other Loans and All Leases |
| 29 | szcrres | Recoveries On Assets Sold and Securitized 1-4 Family Residential Loans |
| 30 | szcrhel | Recoveries On Assets Sold and Securitized Home Equity Lines |
| 31 | szcrcrcd | Recoveries On Assets Sold and Securitized Credit Cards Receivables |
| 32 | szcrauto | Recoveries On Assets Sold and Securitized Auto Loans |
| 33 | szcrcon | Recoveries On Assets Sold and Securitized Other Consumer Loans |
| 34 | szcrci | Recoveries On Assets Sold and Securitized C&I Loans |
| 35 | szcroth | Recoveries On Assets Sold and Securitized All Other Loans and All Leases |
| 36 | asdrres | Assets Sold With Recourse 1-4 Family Residential Loans |
| 37 | asdrhel | Assets Sold With Recourse Home Equity Lines before June 2018 |
| 38 | asdrcrcd | Assets Sold With Recourse Credit Cards Receivables before June 2018 |
| 39 | asdrauto | Assets Sold With Recourse Auto Loans before June 2018 |
| 40 | asdrcons | Assets Sold With Recourse Other Consumer Loans before June 2018 |
| 41 | asdrci | Assets Sold With Recourse C&I Loans before June 2018 |
| 42 | asdroth | Assets Sold With Recourse All Other Loans and All Leases |
| **Maximum Amount of Credit Exposure Retained** | | |
| **Definition** | **Name** | **Label** |
| 1 | SZISLRES | Maximum Credit Exposure 1-4 Residential Securitization |
| 2 | SZISLHEL | Maximum Credit Exposure Home Equity Loan Securitization |
| 3 | SZISLCCD | Maximum Credit Exposure Credit Card Loan Securitization |
| 4 | SZISLAUT | Maximum Credit Exposure Auto Loan Securitization |
| 5 | SZISLCON | Maximum Credit Exposure Consumer Loan Securitization |
| 6 | SZISLCI | Maximum Credit Exposure C&I loan Securitization |
| 7 | SZISLOTH | Max Credit Exposure Other Loan & All Lease Securitization |
| 8 | sziores | Maximum Amount of Credit Exposure, Retained Interest 1-4 Family before June 2018 |
| 9 | sziohel | Maximum Credit Exposure, Retained Interest Home Equity Lines before June 2018 |
| 10 | sziocrcd | Maximum Credit Exposure, Retained Interest Credit Cards Receivables before June |
| 11 | szioauto | Maximum Credit Exposure, Retained Interest Auto Loans before June 2018 |
| 12 | sziocon | Maximum Credit Exposure, Retained Interest Other Consumer Loans before June2018 |
| 13 | szioci | Maximum Credit Exposure, Retained Interest C&I Loans before June 2018 |
| 14 | sziooth | Max Credit Exposure, Retained Interest All Other Lns.&All Lease before June2018 |
| 15 | szsscres | Maximum Credit Exposure,Sub Sec. 1-4 Family Residential Loans before June 2018 |
| 16 | szsschel | Max Credit Exposure, Subordinated Securities Home Equity Lines before June 2018 |
| 17 | szssccrd | Max Credit Exposure, Subordinated Securities Credit Cards Rec. before June 2018 |
| 18 | szsscaut | Max Credit Exposure, Subordinated Securities Auto Loans before June 2018 |
| 19 | szssccon | Max Credit Exposure, Subordinated Securities Other Consumer Lns before June18 |
| 20 | szsscci | Max Credit Exposure, Subordinated Securities C&I before June 2018 |
| 21 | szsscoth | Max Credit Exposure, Subordinated Sec. All Other Lns. & All Lease BeforeJune18 |
| 22 | IDSLCRES | Max Credit Exposure, Standby Letters of Credit 1-4 Family Res. Lns before June18 |
| 23 | IDSLCHEL | Max Credit Exposure, Standby Letters of Credit Home Equity Lines before June2018 |
| 24 | IDSLCCCD | Max Credit Exposure, Standby Letters of Credit Credit Cards Rec. before June2018 |
| 25 | IDSLCAUT | Max Credit Exposure, Standby Letters of Credit Auto Loans before June 2018 |
| 26 | IDSLCCON | Max Credit Exposure, Standby Letters of Credit Other Consumer Lns before June18 |
| 27 | IDSLCCCI | Max Credit Exposure, Standby Letters of Credit C&I Loans before June 2018 |
| 28 | IDSLCOTH | Max Credit Exposure, Standby Letters of Credit All Other Lns.&Ls before June2018 |
| 29 | enceres | Maximum Amount of Credit Exposure, Credit Enhancements 1-4 Family Residential Ln |
| 30 | encehel | Maximum Amount of Credit Exposure, Credit Enhance Home Equity before June 2018 |
| 31 | encecrcd | Maximum Amount of Credit Exp, Credit Enhncmts Credit Cards Recbls before June 18 |
| 32 | enceauto | Maximum Amount of Credit Exposure, Credit Enhancements Auto Loans |
| 33 | encecon | Maximum Amount of Credit Exposure, Credit Enhancements Other Consumer Loans |
| 34 | enceci | Maximum Amount of Credit Exposure, Credit Enhancements C&I Loans |
| 35 | enceoth | Maximum Amount of Credit Exposure, Credit Enhancements All Other Lns. & All Ls. |
| 36 | asceres | Maximum Amount of Credit Exposure, Bank Asset Sales 1-4 Family Residential Loans |
| 37 | ascehel | Maximum Amount of Credit Exposure, Bank Asset Sales Home Equity Lines |
| 38 | ascecrcd | Maximum Amount of Credit Exposure, Bank Asset Sales Credit Cards Receivables |
| 39 | asceauto | Maximum Amount of Credit Exposure, Bank Asset Sales Auto Loans |
| 40 | ascecons | Maximum Amount of Credit Exposure, Bank Asset Sales Other Consumer Loans |
| 41 | asceci | Maximum Amount of Credit Exposure, Bank Asset Sales C&I Loans |
| 42 | asceoth | Maximum Amount of Credit Exposure, Bank Asset Sales All Other Loans & All Leases |
| **Unused Commitments Securitization** | | |
| **Definition** | **Name** | **Label** |
| 1 | szucres | Reporting Bank's Unused Commitments 1-4 Family Residential Loans |
| 2 | szuchel | Reporting Bank's Unused Commitments Home Equity Lines |
| 3 | szuccrcd | Reporting Bank's Unused Commitments Credit Cards Receivables |
| 4 | szucauto | Reporting Bank's Unused Commitments Auto Loans |
| 5 | szuccon | Reporting Bank's Unused Commitments Other Consumer Loans |
| 6 | szucci | Reporting Bank's Unused Commitments C&I Loans |
| 7 | szucoth | Reporting Bank's Unused Commitments All Other Loans and All Leases |
| 8 | ucszres | Reporting Bank's Unused Commitments 1-4 Family Residential Loans |
| 9 | ucszhel | Reporting Bank's Unused Commitments Home Equity Lines before June 2018 |
| 10 | ucszcrcd | Reporting Bank's Unused Commits, Liquidity Credit Cards Recbles before June 18 |
| 11 | ucszauto | Reporting Bank's Unused Commitments, Liquidity Auto Loans |
| 12 | ucszcon | Reporting Bank's Unused Commitments, Liquidity Other Consumer Loans |
| 13 | ucszci | Reporting Bank's Unused Commitments, Liquidity C&I Loans |
| 14 | ucszoth | Reporting Bank's Unused Commitments, Liquidity All Other Lns. & All Ls. |
| **Amount of Ownership (Seller) Interests** | | |
| **Definition** | **Name** | **Label** |
| 1 | ownschel | Amount of Ownership (Seller's), Interest Home Equity Lines |
| 2 | ownsccrd | Amount of Ownership (Seller's), Interest Credit Cards Receivables |
| 3 | ownscci | Amount of Ownership (Seller's), Interest C&I Loans |
| 4 | ownp3hel | Past Due Loan Amounts 30-89 Days Home Equity Lines |
| 5 | ownp3crd | Past Due Loan Amounts 30-89 Credit Cards Receivables |
| 6 | ownp3ci | Past Due Loan Amounts 30-89 Days C&I Loans |
| 7 | ownp9hel | Past Due Loan Amounts 90+ Days Home Equity Lines |
| 8 | ownp9crd | Past Due Loan Amounts 90+ Days Credit Cards Receivables |
| 9 | ownp9ci | Past Due Loan Amounts 90+ Days C&I Loans |
| 10 | owndrhel | Charge-Offs On Loan Amounts |
| 11 | owndrcrd | Charge-Offs On Loan Amounts Credit Cards Receivables |
| 12 | owndrci | Charge-Offs On Loan Amounts C&I Loans |
| 13 | owncrhel | Recoveries On Loan Amounts Home Equity Lines |
| 14 | owncrcrd | Recoveries On Loan Amounts Credit Cards Receivables |
| 15 | owncrci | Recoveries On Loan Amounts C&I Loans |
| 16 | ownlnhel | Amount of Ownership (Seller's), Interest Carried as Loans Home Equity Lines |
| 17 | ownlncrd | Amount of Ownership (Seller's), Interest Carried as Loans Credit Cards Rec. |
| 18 | ownlnci | Amount of Ownership (Seller's) Interest, Carried as Loans C&I Loans |
| **Memoranda** | | |
| **Definition** | **Name** | **Label** |
| 1 | lnsb | Outstanding principal balance of obligations transferred |
| 2 | lnsbr | Amount of retained recourse exposure |
| 3 | msrece | Outstanding Princiapl Balance of Assets Serviced for Others 1-4 Family Res. Mrtg |
| 4 | msrnrece | Outstanding Princiapl Balance of Assets Serviced for Others 1-4 Family Res. Mrtg |
| 5 | lnserv | Outstanding Princiapl Balance of Assets Serviced for Others Other Fin. Assets |
| 6 | MSRESFCL | Serviced 1-4 family loans in foreclosure |
| 7 | abcxbk | Asset-Backed Commercial Paper Conduits Sponsored by the Bank |
| 8 | abcxoth | Asset-Backed Commercial Paper Conduits Sponsored by Other Unrelated Inst. |
| 9 | abcubk | Unused Commitments to Provide Liquidity to Conduit Structures |
| 10 | abcuoth | Unused Commitments to Provide Liquidity, Unrelated Institutions |
| 11 | szcrcdfe | Outstanding Credit Card Fees and Financial Charges |
| **Income and Expense** | | |
| **Definition** | **Name** | **Label** |
| 1 | intinc/intinq | Total interest income |
| 2 | eintexp/eintxq | Total interest expense |
| 3 | nim/nimq | Net interest income |
| 4 | elnatr/elnatq | Provision for loan and lease losses |
| 5 | nonii/noniiq | Total noninterest income |
| 6 | ifiduc/ifiducq | Gross Fiduciary activities income |
| 7 | iserchg/iserchgq | Service charges on deposit accounts |
| 8 | igltrad/igltrdq | Trading account gains and fees |
| 9 | idothnii/idothniq | Additional Noninterest Income |
| 10 | nonix/nonixq | Total noninterest expense |
| 11 | esal/esalq | Salaries and employee benefits |
| 12 | epremagg/epremagq | Premises and equipment expense |
| 13 | IDEOTH/IDEOTHQ | Additional noninterest expense |
| 14 | idpretx/idpretxq | Pre-tax net operating income |
| 15 | iglsec/iglsecq | Securities gains (losses) |
| 16 | itax/itaxq | Applicable income taxes |
| 17 | ibefxtr/ibefxtrq | Income before extraordinary items |
| 18 | extra/extraq | Extraordinary gains, net |
| 19 | netinc/netincq | Net income |
| 20 | NETIMIN/NETIMINQ | Minority interest net income |
| 21 | NETINBM/NETINBMQ | Net income of bank and minority interests. |
| 22 | ntlnls/ntlnlsq | Net charge-offs |
| 23 | eqcdiv/eqcdivq | Cash dividends |
| 24 | eqcstkrx/idnull | Sale, conversion, retirement of capital stock, net |
| 25 | noij/noijq | Net operating income |
| **Total Interest Income** | | |
| **Definition** | **Name** | **Label** |
| 1 | intinc/intinq | Total interest income |
| 2 | ilndom/ilndomq | Interest income: Domestic office loans |
| 3 | ilnfor/ilnforq | Interest income: Foreign office loans |
| 4 | ils/ilsq | Interest income: Lease financing receivables |
| 5 | ichbal/ichbalq | Interest Income: Balance due from depository inst |
| 6 | isc/iscq | Interest income: Securities |
| 7 | itrade/itradeq | Interest income: Trading accounts |
| 8 | ifrepo/ifrepoq | Interest income: Federal funds sold |
| 9 | IOTHII/IOTHIIQ | Other interest income |
| **Total Interest Expense** | | |
| **Definition** | **Name** | **Label** |
| 1 | eintexp/eintxq | Total interest expense |
| 2 | edepdom/edepdomq | Interest expense: Domestic office deposits |
| 3 | edepfor/edepforq | Interest expense: Foreign office deposits |
| 4 | efrepp/efreppq | Interest expense: Federal funds purchased |
| 5 | ettlotmg/ETTLOTMQ | Interest Expense:Demand notes, US treasury & other |
| 6 | IDEOTHINT/IDEOTHINTQ | Other interest expense |
| 7 | esubnd/esubndq | Interest Expense: Subordinated notes and debenture |
| **Trading Account Gains & Fees** | | |
| **Definition** | **Name** | **Label** |
| 1 | igltrad | Trading account gains and fees |
| 2 | iglrtex | Interest rate exposures |
| 3 | iglfxex | Foreign exchange exposures |
| 4 | igledex | Equity security and index exposures |
| 5 | iglcmex | Commondity and other exposures |
| 6 | IGLCREX | Trading revenues on credit exposures |
| **Additional Noninterest Income** | | |
| **Definition** | **Name** | **Label** |
| 1 | idothnii/idothniq | Additional Noninterest Income |
| 2 | iinvfee/iinvfeeq | Investment banking, advisory, brokerage, and underwriting fees and commissions |
| 3 | ivencap/ivencapq | Venture capital revenue |
| 4 | iserfee/iserfeeq | Net servicing fees |
| 5 | isecz/iseczq | Net securitization income |
| 6 | iinscom/iinscomq | Insurance commission fees and income |
| 7 | iinsund/iinsundq | Insurance underwriting income |
| 8 | iinsoth/iinsothq | Other insurance commissions and fees |
| 9 | netgnsln/ntgllnq | Net gains (losses) on sales of loans |
| 10 | netgnsre/ntglreq | Net gains (losses) on sales of other real estate owned |
| 11 | netgnast/ntglfxaq | Net gains (losses) on sales of other assets (excluding securities) |
| 12 | iotnii/iotniiq | Other non-interest income |
| **Additional Noninterest Expense** | | |
| **Definition** | **Name** | **Label** |
| 1 | IDEOTH/ideothq | Additional noninterest expense |
| 2 | EAMINTAN/EAMINTQ | Goodwill impairment losses and amortization expense |
| 3 | EINTOTH/EINTOTHQ | Amortization expense and impairment losses for other intangible losses |
| 4 | EINTGW/EINTGWQ | Goodwill impairment losses |
| 5 | eothnint/eothninq | All other noninterest expense |
| **Loan Charge-Offs and Recoveries** | | |
| **Definition** | **Name** | **Label** |
| 1 | drlnls/drlnlsq | Total charge-offs |
| 2 | drre/drreq | Loans secured by real estate, total |
| 3 | drrenus/idnull | To non-U.S. addressees |
| 4 | iddrredm/iddrredq | Real estate loans in domestic offices |
| 5 | drrecons/drreconq | Construction and land development |
| 6 | DRRECNFM/DRRECNFQ | 1-4 family residential construction loan charge offs |
| 7 | DRRECNOT/DRRECNOQ | Other construction loans and all land development and other land loan charge off |
| 8 | drreag/drreagq | Secured by farmland |
| 9 | drreres/drreresq | Total charge-offs secured by 1-4 family residential properties |
| 10 | drremult/drremulq | Secured by multifamily residential properties |
| 11 | drrenres/drrenrsq | Secured by nonfarm nonresidential properties |
| 12 | DRRENROW/DRRENOWQ | Secured by owner-occupied nonfarm nonresidential properties - charge offs |
| 13 | DRRENROT/DRRENOTQ | Secured by other nonfarm nonresidential properties - charge offs |
| 14 | drrefor/drreforq | Real estate loans in foreign offices |
| 15 | drdep/drdepq | Loans to depository institutions |
| 16 | drdepnus/idnull | To foreign banks |
| 17 | drag/dragq | Loans to finance agricultural production and other loans to farmers |
| 18 | drci/drciq | Commercial and industrial loans |
| 19 | drcon/drconq | Loans to individuals |
| 20 | drcrcd/drcrcdq | Credit card loan charge offs |
| 21 | DRAUTO/DRAUTOQ | Auto Loans Charge-Offs |
| 22 | DRCONOTH/DRCONOTQ | Other Consumer Loan Charge-Offs |
| 23 | drforgv/drforgvq | Loans to foreign governments and official institutions |
| 24 | drother/drothq | Other loans |
| 25 | drls/drlsq | Lease financing receivables |
| 26 | dragsm/dragsmq | Loans to finance agricultural production and other loans to farmers |
| 27 | crlnls/crlnlsq | Total recoveries |
| 28 | crre/crreq | Loans secured by real estate, total |
| 29 | crrenus/idnull | To non-U.S. addressees |
| 30 | idcrredm/idcrredq | Real estate loans in domestic offices |
| 31 | crrecons/crreconq | Construction and land development |
| 32 | CRRECNFM/CRRECNFQ | 1-4 family residential construction loan recoveries |
| 33 | CRRECNOT/CRRECNOQ | Other construction loans and all land development and other land loan recoveries |
| 34 | crreag/crreagq | Secured by farmland |
| 35 | crreres/crreresq | Secured by 1-4 family residential properties - total recoveries |
| 36 | crremult/crremulq | Secured by multifamily residential properties |
| 37 | crrenres/crrenrsq | Secured by nonfarm nonresidential properties |
| 38 | CRRENROW/CRRENOWQ | Secured by owner-occupied nonfarm nonresidential property - recoveries |
| 39 | CRRENROT/CRRENOTQ | Secured by other nonfarm nonresidential property - recoveries |
| 40 | crrefor/crreforq | Real estate loans in foreign offices |
| 41 | crdep/crdepq | Loans to depository institutions |
| 42 | crdepnus/idnull | To foreign banks |
| 43 | crag/cragq | Loans to finance agricultural production and other loans to farmers |
| 44 | crci/crciq | Commercial and industrial loans |
| 45 | crcon/crconq | Loans to individuals |
| 46 | crcrcd/crcrcdq | Credit card loan recoveries |
| 47 | CRAUTO/CRAUTOQ | Auto Loans - Recoveries |
| 48 | CRCONOTH/CRCONOTQ | Other Consumer Loan Recoveries |
| 49 | crforgv/crforgvq | Loans to foreign governments and official institutions |
| 50 | crother/crothq | Other loans |
| 51 | crls/crlsq | Lease financing receivables |
| 52 | cragsm/cragsmq | Loans to finance agricultural production and other loans to farmers |
| 53 | ntlnls/ntlnlsq | Net charge-offs |
| 54 | ntre/ntreq | Loans secured by real estate, total |
| 55 | ntrenus/idnull | To non-U.S. addressees |
| 56 | idntredm/idntredq | Real estate loans in domestic offices |
| 57 | ntrecons/ntreconq | Construction and land development |
| 58 | NTRECNFM/NTRECNFQ | 1-4 family residential construction loan net charge offs |
| 59 | NTRECNOT/NTRECNOQ | Other contruction loans and all land development and other land loan net charge |
| 60 | ntreag/ntreagq | Secured by farmland |
| 61 | ntreres/ntreresq | Secured by 1-4 family residential properties� net charge-offs |
| 62 | ntremult/ntremulq | Secured by multifamily residential properties |
| 63 | ntrenres/ntrenrsq | Secured by nonfarm nonresidential properties |
| 64 | NTRENROW/NTRENOWQ | Secured by owner-occupied nonfarm nonresidential properties - net charge offs |
| 65 | NTRENROT/NTRENOTQ | Secured by other nonfarm nonresidential properties - net charge offs |
| 66 | ntrefor/ntreforq | Real estate loans in foreign offices |
| 67 | ntdep/ntdepq | Loans to depository institutions |
| 68 | ntdepnus/idnull | To foreign banks |
| 69 | ntag/ntagq | Loans to finance agricultural production and other loans to farmers |
| 70 | ntci/ntciq | Commercial and industrial loans |
| 71 | ntcinus/idnull | To non-U.S. addressees |
| 72 | ntcon/ntconq | Loans to individuals |
| 73 | ntcrcd/ntcrcdq | Credit card loan net charge offs |
| 74 | NTAUTO/NTAUTOQ | Auto Loans - Net Charge-Offs |
| 75 | NTCONOTH/NTCONOTQ | Other Consumer Loans Net Charge-Offs |
| 76 | ntforgv/ntforgvq | Loans to foreign governments and official institutions |
| 77 | ntother/ntothq | Other loans |
| 78 | ntls/ntlsq | Lease financing receivables |
| 79 | ntagsm/ntagsmq | Loans to finance agricultural production and other loans to farmers |
| **Total Charge-offs 1-4 Family Residential** | | |
| **Definition** | **Name** | **Label** |
| 1 | drreres/drreresq | Total charge-offs secured by 1-4 family residential properties |
| 2 | DRRERSFM/idnull | Loans secured by 1-4 family senior liens � total charge-offs |
| 3 | DRRERSF2/idnull | Loans secured by 1-4 family junior liens � total charge-offs |
| 4 | drreloc/drrelocq | Total charge offs - home equity lines of credit |
| **Total Recoveries 1-4 Family Residential** | | |
| **Definition** | **Name** | **Label** |
| 1 | crreres/crreresq | Secured by 1-4 family residential properties - total recoveries |
| 2 | CRRERSFM/idnull | Loans secured by 1-4 family senior liens � total recoveries |
| 3 | CRRERSF2/idnull | Loans secured by 1-4 family junior liens � total recoveries |
| 4 | crreloc/crrelocq | Home equity lines of credit - total recoveries |
| **Net Charge-offs 1-4 Family Residential** | | |
| **Definition** | **Name** | **Label** |
| 1 | ntreres/ntreresq | Secured by 1-4 family residential properties� net charge-offs |
| 2 | NTRERSFM/NTRERSFQ | Loans secured by 1-4 family senior liens � net charge-offs |
| 3 | NTRERSF2/NTRERS2Q | Loans secured by 1-4 family junior liens � net charge-offs |
| 4 | ntreloc/ntrelocq | Home equity lines of credit � net charge-offs |
| **Cash Dividends** | | |
| **Definition** | **Name** | **Label** |
| 1 | eqcdiv | Cash dividends |
| 2 | eqcdivp | Cash dividends declared on preferred stock |
| 3 | eqcdivc | Cash dividends declared on common stock |
| **Interest income and expense in foreign offices** | | |
| **Definition** | **Name** | **Label** |
| 1 | ilnfor | Interest income: Foreign office loans |
| 2 | edepfor | Interest expense: Foreign office deposits |
| **Performance and Condition Ratios** | | |
| **Definition** | **Name** | **Label** |
| 1 | idntilr/IDNTILRQ | % of unprofitable institutions |
| 2 | idntigr/idntigrq | % of insitutions with earnings gains |
| 3 | intincy/intincyq | Yield on earning assets |
| 4 | intexpy/intexpyq | Cost of funding earning assets |
| 5 | nimy/nimyq | Net interest margin |
| 6 | noniiay/noniiayq | Noninterest income to average assets |
| 7 | nonixay/nonixayq | Noninterest expense to average assets |
| 8 | ELNATRY/ELNATRYQ | Loan and lease loss provision to assets |
| 9 | noijy/noijyq | Net operating income to assets |
| 10 | roa/roaq | Return on assets (ROA) |
| 11 | roaptx/roaptxq | Pretax return on assets |
| 12 | roe/roeq | Return on Equity (ROE) |
| 13 | roeinjr/roeinjrq | Retained earnings to average equity (ytd only) |
| 14 | ntlnlsr/ntlnlsqr | Net charge-offs to loans |
| 15 | elnantr/elnatqr | Credit loss provision to net charge-offs |
| 16 | iderncvr/iderncvq | Earnings coverage of net charge-offs (x) |
| 17 | eeffr/eeffqr | Efficiency ratio |
| 18 | astempm/astempm | Assets per employee ($millions) |
| 19 | iddivnir/iddivnir | Cash dividends to net income (ytd only)\* |
| 20 | ERNASTR/ERNASTR | Earning assets to total assets ratio |
| 21 | lnatresr/lnatresr | Loss allowance to loans |
| 22 | lnresncr/lnresncr | Loan loss allowance to noncurrent loans |
| 23 | nperfv/nperfv | Noncurrent assets plus other real estate owned to assets |
| 24 | nclnlsr/nclnlsr | Noncurrent loans to loans |
| 25 | LNLSNTV/LNLSNTV | Net loans and leases to total assets |
| 26 | lnlsdepr/lnlsdepr | Net loans and leases to deposits |
| 27 | idlncorr/idlncorr | Net loans and leases to core deposits |
| 28 | DEPDASTR/DEPDASTR | Total domestic deposits to total assets |
| 29 | eqv/eqv | Equity capital to assets |
| 30 | rbc1aaj/rbc1aaj | Core capital (leverage) ratio |
| 31 | rbc1rwaj/rbc1rwaj | Tier 1 risk-based capital ratio |
| 32 | rbcrwaj/rbcrwaj | Total risk-based capital ratio |
| 33 | RBCT1CER/RBCT1CER | Common equity tier 1 capital ratio |
| 34 | asset5/asset2 | Average total assets |
| 35 | ernast5/ernast2 | Average earning assets |
| 36 | eq5/eq2 | Average equity |
| 37 | LNLSGR5/LNLSGR2 | Average total loans |
| **Net charge-offs to loans** | | |
| **Definition** | **Name** | **Label** |
| 1 | ntlnlsr/ntlnlsqr | Net charge-offs to loans |
| 2 | ntrer/ntreqr | % Net Loans Charged-off: Total real estate loans |
| 3 | ntrecosr/ntrecoqr | % Net Loans Charged-off:Construction & development |
| 4 | ntrenrsr/NTRENRQR | % Net Loans Charged-off: Commercial real estate |
| 5 | ntremulr/ntremuqr | % Net Loans Charged-off: Multi-family residential |
| 6 | ntreresr/idntrrqr | % Net Loans Charged-off: 1-4 family residential |
| 7 | ntrelocr/idntrlqr | % Net Loans Charged-off: Home equity loans |
| 8 | ntreothr/idntrorq | All other 1-4 family - Percent of loans charged-off, net |
| 9 | idntcir/idntciqr | % Net Loans Charged-off: Commercial and industrial |
| 10 | idntconr/idntcnqr | % Net Loans Charged-off: Loans to individuals |
| 11 | idntcrdr/idntcdqr | % Net Loans Charged-off: Credit card loans |
| 12 | idntcoor/idntcoqr | % Net Loans Charged-off: Other loans to individual |
| 13 | idntator/NTAUTOQR | % Net Loans Charged-off: Automobile loans |
| 14 | idntcotr/NTCONTQR | % Net Loans Charged-off: Other Consumer Loans |
| 15 | ntcomrer/idntcmqr | % Net Loans Charged-off:Coml. RE not secured by RE |
| **Noncurrent loans to loans** | | |
| **Definition** | **Name** | **Label** |
| 1 | nclnlsr/ | Noncurrent loans to loans |
| 2 | ncrer/ | % Loans Noncurrent: Real estate loans |
| 3 | ncreconr/ | % Loans Noncurrent:Construction & land development |
| 4 | ncrenrer/ | % Loans Noncurrent: Commercial real estate |
| 5 | ncremulr/ | % Loans Noncurrent: Multifamily residential |
| 6 | ncreresr/ | % Loans Noncurrent: 1-4 family residential |
| 7 | ncrelocr/ | % Loans Noncurrent: Home equity loans |
| 8 | ncrereor/ | Percent of loans noncurrent - All other family |
| 9 | idnccir/ | % Loans noncurrent:Commercial and industrial loans |
| 10 | idncconr/ | % Loans Noncurrent: Loans to individuals |
| 11 | idnccrdr/ | % Loans Noncurrent: Credit card loans |
| 12 | idnccoor/ | % Loans Noncurrent: Other loans to individuals |
| 13 | IDNCATOR/ | % Loans Noncurrent: Automobile loans |
| 14 | IDNCCOTR/ | % Loans Noncurrent: Other consumer loans |
| 15 | nccomrer/ | % Loans Noncurrent:Commercial RE not secured by RE |
| 16 | idncgtpr/ | Wholly or partially US Gov. guaranteed noncurrent loans as percent of noncurrent |
| **Demographics Info** | | |
| **Definition** | **Name** | **Label** |
| 1 | cert | FDIC Certificate # |
| 2 | docket | OTS Docket Number |
| 3 | fed\_rssd | Federal Reserve ID Number |
| 4 | rssdhcr | RSSDID Number |
| 5 | name | Institution name |
| 6 | city | City |
| 7 | state | State |
| 8 | zip | Zip |
| 9 | repdte | Report Date |
| 10 | rundate | Run Date |
| 11 | bkclass | Bank Charter Class |
| 12 | Address | Physical Street Address |
| 13 | namehcr | Bank Holding Company (Regulatory Top Holder) |
| 14 | offdom | Number of Domestic U.S. Offices |
| 15 | offfor | Number of Foreign Offices |
| 16 | stmult | Interstate Branches |
| 17 | specgrp | Asset Concentration Hierarchy |
| 18 | subchaps | Subchapter S Corporations |
| 19 | county | County |
| 20 | CBSA\_Metro | MSA number based on 2000 Census |
| 21 | CBSA\_Metro\_name | MSA name based on 2000 Census |
| 22 | estymd | Established Date |
| 23 | insdate | Date of Deposit Insurance |
| 24 | effdate | Last Structure Change Effective Date |
| 25 | mutual | Mutual Ownership Flag |
| 26 | parcert | Directly owned by another bank (CERT) |
| 27 | trust | Trust Powers |
| 28 | regagnt | Regulator |
| 29 | insagnt1 | Insurance Fund Membership |
| 30 | fdicdbs | FDIC Regions |
| 31 | fdicsupv | FDIC Supervisory Region |
| 32 | fldoff | FDIC Field Office |
| 33 | fed | Federal Reserve District |
| 34 | occdist | Office of the Comptroller District |
| 35 | otsregnm | Office of Thrift Supervision Region - Before July 21, 2011 |
| 36 | offoa | Number of Offices in Insured Other Areas |
| 37 | cb | FDIC Community Banks |
| 38 | webaddr | Primary Internet Web Address: |
|  | | |
|  | | |
|  | | |
| The large download file is created by sdiLargeDownload Utility (Version: 3.0.3) | | |
