# Supplementary material for: Panel and geospatial data for U.S. FDIC insured banks fiduciary activities and annual performance analyses over the periods 2016 to 2018
Source: Data Brief. 2019 Aug 6;25:104358. doi: 10.1016/j.dib.2019.104358 (PMC6704373; doi:10.1016/j.dib.2019.104358)
Supplement: Supplementary file 1 [file mmc1.zip › R data and Codes Supplements/0-Initial Panel data/Raw Excel Format FDIC banks reporting Data/Combined Data sets/Variables definition.docx]

| **Bank Charter Class : bkclass** |
| --- |
| A classification code assigned by the FDIC based on the institution's charter type (commercial bank or savings institution), charter agent (state or federal), Federal Reserve membership status (Fed member, Fed nonmember)and its primary federal regulator (state chartered institutions are subject to both federal and state supervision).   - **N** = commercial bank, national (federal) charter and Fed member, supervised by the Office of the Comptroller of the Currency (OCC) - **SM** = commercial or savings bank, state charter and Fed member, supervised by the Federal Reserve (FRB) - **NM** = commercial bank, state charter and Fed nonmember, supervised by the FDIC or OCC - **SB** = savings banks, state charter, supervised by the FDIC - **SA** = As of July 21, 2011, FDIC supervised state chartered thrifts and OCC supervised federally chartered thrifts. Prior to that date, state or federally chartered savings associations supervised by the Office of Thrift Supervision (OTS). - **OI** = insured U.S. branch of a foreign chartered institution (IBA) |

| **Number of Domestic U.S. Offices: offdom** |
| --- |
| The number of domestic offices (including headquarters) in the 50 states of the U.S.A. operated by active institutions. |

| **Number of Foreign Offices : offfor** |
| --- |
| The number of foreign offices (outside the U.S. and U.S.territories) operated by active FDIC Insured institutions. |

| **Interstate Branches : stmult** |
| --- |
| A 'yes' indicates that an institution has branches that can accept FDIC-insured deposits in more than one state.  This item is not available (NA) for [TFR Reporters](https://www5.fdic.gov/sdi/definitions.asp?SystemForm=ID&HelpItem=tfrrpt).  The FDIC Act defines state as any State of the United States, the District of Columbia, and any territory of the United States, Puerto Rico, Guam, American Samoa, the Trust Territory of the Pacific Islands, the Virgin Island, and the Northern Mariana Islands. |

| **Asset Concentration Hierarchy : specgrp** |
| --- |
| An indicator of an institution�s primary specialization in terms of asset concentration.   **Note: (Groups are hierarchical and mutually exclusive):**  **1 - International Specialization**� Institutions with assets greater than $10 billion and more than 25 percent of total assets in foreign offices.  **2 - Agricultural Specialization**� Banks with agricultural production loans plus real estate loans secured by farmland in excess of 25 percent of total loans and leases.  **3 - Credit-card Specialization**� Institutions with credit-card loans plus securitized receivables in excess of 50 percent of total assets plus securitized receivables.  **4 - Commercial Lending Specialization**� Institutions with commercial and industrial loans, plus real estate construction and development loans, plus loans secured by commercial real estate properties in excess of 25 percent of total assets.  **5 - Mortgage Lending Specialization**� Institutions with residential mortgage loans, plus mortgage-backed securities, in excess of 50 percent of total assets.  **6 - Consumer Lending Specialization**� Institutions with residential mortgage loans, plus credit-card loans, plus other loans to individuals, in excess of 50 percent of total assets.  **7 - Other Specialized < $1 Billion**� Institutions with assets less than $1 billion and with loans and leases are less than 40 percent of total assets.  **8 - All Other < $1 Billion**� Institutions with assets less than $1 billion that do not meet any of the definitions above, they have significant lending activity with no identified asset concentrations.  **9 - All Other > $1 Billion**� Institutions with assets greater than $1 billion that do not meet any of the definitions above, they have significant lending activity with no identified asset concentrations. |
| **FDIC Regions**: fdicdbs |
| The six geographic FDIC Regions and their respective states are:   - New York (02) � States: Connecticut, Delaware, Maine, Maryland, Massachusetts, New Hampshire, New Jersey, New York, Pennsylvania, Puerto Rico, Rhode Island, Vermont, Virgin Islands - Atlanta (05) - States: Alabama, Florida, Georgia, North Carolina, South Carolina, Virginia - Chicago (09)�States: Illinois, Indiana, Kentucky, Michigan, Ohio - Kansas City (11) � States: Iowa, Kansas, Minnesota, Missouri, Nebraska, North Dakota, South Dakota - Dallas (13)- States: Arkansas, Colorado, Louisiana, Mississippi, New Mexico, Oklahoma, Tennessee, Texas - San Francisco (14)�States: Alaska, American Samoa, Arizona, California, Federated States of Micronesia, Guam, Hawaii, Idaho, Montana, Nevada, Oregon, Utah, Washington, Wyoming |

All Above Variables are descriptive of FDIC insured Banks Demographic Information for use as background controls

Performance and Condition Ratios:

1. Dependent variables

| **Yield on earning assets : intincy** |
| --- |
| Total interest income (annualized) as a percent of [average earning assets](https://www5.fdic.gov/sdi/definitions.asp?SystemForm=ID&HelpItem=ernast5)**.** |
| **Average earning assets** |
| The average of all loans and other investments that earn interest or dividends. Averages are calculated as follows:   - **Year-to-date averages:**   - **March reporting period** - (December earning assets + March earning assets) / 2   - **June reporting period** - (December earning assets + March earning assets + June earning assets) / 3   - **September reporting period** - (December earning assets + March earning assets + June earning assets + September earning assets) / 4   - **December reporting period** - (Previous December earning assets + March earning assets + June earning assets + September earning assets + December earning assets) / 5 |

| **Return on assets (ROA) : roa** |
| --- |
| Net income after taxes and extraordinary items (annualized) as a percent of [average total assets](https://www5.fdic.gov/sdi/definitions.asp?SystemForm=ID&HelpItem=asset5).   \| **Average total assets** \| \| --- \| \| Year-to-date average of the total assets represented on the balance sheet. Used as the denominator for year-to-date income as a percent of average assets. The number of quarterly values used in the calculation depends on the date of the data.   - **Year-to-date averages:**   - **March reporting period** - (December assets + March assets) / 2   - **June reporting period** - (December assets + March assets + June assets) / 3   - **September reporting period** - (December assets + March assets + June assets + September assets) / 4   - **December reporting period** - (Previous December assets + March assets + June assets + September assets + December assets) / 5 \| |

| **Return on Equity (ROE) : roe** |
| --- |
| Annualized net income as a percent of [average total equity](https://www5.fdic.gov/sdi/definitions.asp?SystemForm=ID&HelpItem=eq5) on a consolidated basis. Note: If retained earnings are negative, the ratio is shown as NA   \| **Average equity** \| \| --- \| \| The average of total equity capital (includes preferred and common stock, surplus and undivided profits). Averages are calculated as follows:   - **Year-to-date averages:**   - **March reporting period** - (December equity + March equity) / 2   - **June reporting period** - (December equity + March equity + June equity) / 3   - **September reporting period** - (December equity + March equity + June equity + September equity) / 4   - **December reporting period** - (Previous December equity + March equity + June equity + September equity + December equity) / 5 \| |
| **Efficiency ratio**: eeffr |
| Noninterest expense less amortization of intangible assets as a percent of net interest income plus noninterest income. This ratio measures the proportion of net operating revenues that are absorbed by overhead expenses, so that a lower value indicates greater efficiency. |

| **Core capital (leverage) ratio : rbc1aaj** |
| --- |
| Tier 1 (core) capital as a percent of average total assets minus ineligible intangibles.  Tier 1 (core) capital includes: common equity plus noncumulative perpetual preferred stock plus minority interests in consolidated subsidiaries less goodwill and other ineligible intangible assets. The amount of eligible intangibles (including mortgage servicing rights) included in core capital is limited in accordance with supervisory capital regulations. Average total assets used in this computation are an average of daily or weekly figures for the quarter.  As of March 2015, all institutions began reporting the amended CALL schedule RC-R Part I and Part II which incorporates risk-based capital rules based on the Basel III framework and section 939A of the Dodd-Frank Act. Some designated institutions began reporting based on the updated requirements as of March 2014. (See: FDIC Financial Institutions Letter FIL-24-2012) |

| **Tier 1 risk-based capital ratio: rbc1rwaj** |
| --- |
| Tier 1 (core) capital as a percent of risk-weighted assets as defined by the appropriate [federal regulator](https://www5.fdic.gov/sdi/HelpItemForm.asp?SystemForm=ID&HelpItem=regagnt) for prompt corrective action during that time period.  As of March 2015, all institutions began reporting the amended CALL schedule RC-R Part I and Part II which incorporates risk-based capital rules based on the Basel III framework and section 939A of the Dodd-Frank Act. Some designated institutions began reporting based on the updated requirements as of March 2014. (See: FDIC Financial Institutions Letter FIL-24-2012) |

| **Total risk-based capital ratio: rbcrwaj** |
| --- |
| Total risk based capital as a percent of risk-weighted assets as defined by the appropriate [federal regulator](https://www5.fdic.gov/sdi/HelpItemForm.asp?SystemForm=ID&HelpItem=regagnt) for prompt corrective action during that time period.  As of March 2015, all institutions began reporting the amended CALL schedule RC-R Part I and Part II which incorporates risk-based capital rules based on the Basel III framework and section 939A of the Dodd-Frank Act. Some designated institutions began reporting based on the updated requirements as of March 2014. (See: FDIC Financial Institutions Letter FIL-24-2012) |

1. Explanatory Variables

| **Net interest margin : nimy** |
| --- |
| Total interest income less total interest expense (annualized) as a percent of [average earning assets](https://www5.fdic.gov/sdi/definitions.asp?SystemForm=ID&HelpItem=ernast5).   \| **Average earning assets** \| \| --- \| \| The average of all loans and other investments that earn interest or dividends. Averages are calculated as follows:   - **Year-to-date averages:**   - **March reporting period** - (December earning assets + March earning assets) / 2   - **June reporting period** - (December earning assets + March earning assets + June earning assets) / 3   - **September reporting period** - (December earning assets + March earning assets + June earning assets + September earning assets) / 4   - **December reporting period** - (Previous December earning assets + March earning assets + June earning assets + September earning assets + December earning assets) / 5 \| |

| **Net operating income to assets: noijy** |
| --- |
| Net operating income (annualized) as a percent of [average total assets](https://www5.fdic.gov/sdi/definitions.asp?SystemForm=ID&HelpItem=asset5)   \| **Average total assets** \| \| --- \| \| Year-to-date average of the total assets represented on the balance sheet. Used as the denominator for year-to-date income as a percent of average assets. The number of quarterly values used in the calculation depends on the date of the data.   - **Year-to-date averages:**   - **March reporting period** - (December assets + March assets) / 2   - **June reporting period** - (December assets + March assets + June assets) / 3   - **September reporting period** - (December assets + March assets + June assets + September assets) / 4   - **December reporting period** - (Previous December assets + March assets + June assets + September assets + December assets) / 5 \| |

| **Net charge-offs to loans: ntlnlsr** |
| --- |
| Gross loan and lease financing receivable charge-offs, less gross recoveries, (annualized) as a percent of [average total loans and lease financing receivables](https://www5.fdic.gov/sdi/definitions.asp?SystemForm=ID&HelpItem=lnlsgr5).   \| **Average total loans** \| \| --- \| \| The average of total loans and lease financing receivables, net of unearned income. Averages are calculated as follows:   - **Year-to-date averages:**   - **March reporting period** - (December total loans + March total loans) / 2   - **June reporting period** - (December total loans + March total loans + June total loans) / 3   - **September reporting period** - (December total loans + March total loans + June total loans + September total loans) / 4   - **December reporting period** - (Previous December total loans + March total loans + June total loans + September total loans + December total loans) / 5 \| |

| **Credit loss provision to net charge-offs: elnantr** |
| --- |
| Provision for possible credit and allocated transfer risk as a percent of net charge-offs. If the denominator is less than or equal to zero, then ratio is shown as "NA." |

| **Earnings coverage of net charge-offs (x): ernastr** |
| --- |
| Income before income taxes and extraordinary items and other adjustments, plus provisions for loan and lease losses and allocated transfer risk reserve, plus gains (losses) on securities not held in trading accounts (annualized) divided by net loan and lease charge-offs (annualized). This is a number of times ratio (x) not a percentage ratio (%).  ***** If the denominator is less than or equal to zero, then ratio is shown as "N/A.  RIS definition:  YR - IDERNCVR = CHFLA / NTLNLSA QTR - IDERNCVQ = CHFLQ / NTLNLSQ |

| **Assets per employee ($millions) : astempm** |
| --- |
| Total assets in millions of dollars as a percent of the number of full-time equivalent employees. |

| **Earning assets to total assets ratio: ernastr** |
| --- |
| Interest earning assets as a percent of total assets. |

| **Net loans and leases to total assets: lnlsntv** |
| --- |
| Loan and lease financing receivables, net of unearned income, allowances, and reserves, as a percent of total assets. |

| **Total domestic deposits to total assets: depdastr** |
| --- |
| Total domestic office deposits as a percent of total assets. |

| **Equity capital to assets : eqv** |
| --- |
| Total equity capital as a percent of total assets. |

| **Average total assets : asset5** |
| --- |
| Year-to-date average of the total assets represented on the balance sheet. Used as the denominator for year-to-date income as a percent of average assets. The number of quarterly values used in the calculation depends on the date of the data.   - **Year-to-date averages:**   - **March reporting period** - (December assets + March assets) / 2   - **June reporting period** - (December assets + March assets + June assets) / 3   - **September reporting period** - (December assets + March assets + June assets + September assets) / 4   - **December reporting period** - (Previous December assets + March assets + June assets + September assets + December assets) / 5 |

| **Average earning assets : ernast5** |
| --- |
| The average of all loans and other investments that earn interest or dividends. Averages are calculated as follows:   - **Year-to-date averages:**   - **December reporting period** - (Previous December earning assets + March earning assets + June earning assets + September earning assets + December earning assets) / 5 |
|  |
| **Average equity : eq5** |
| The average of total equity capital (includes preferred and common stock, surplus and undivided profits). Averages are calculated as follows:   - **Year-to-date averages:**   - **December reporting period** - (Previous December equity + March equity + June equity + September equity + December equity) / 5 |
|  |
| **Average total loans : lnlsgr5** |
| The average of total loans and lease financing receivables, net of unearned income. Averages are calculated as follows:   - **Year-to-date averages:**   - **December reporting period** - (Previous December total loans + March total loans + June total loans + September total loans + December total loans) / 5 |

Primary variables of interest: They define U.S. FDIC insured banks annual fiduciary activities

| **Fiduciary powers granted : trpower** |
| --- |
| Institution has been **granted** trust powers by a state or national regulatory authority to administer accounts in a fiduciary capacity. Fiduciary capacity generally means: trustee, executor, administrator, registrar of stocks and bonds, transfer agent, guardian, assignee, receiver, custodian under a uniform gifts to minors act, investment advisor, any capacity in which the institution possesses investment discretion on behalf of another, or any similar capacity.   \| **Note:** \| Domestic offices of foreign banks (a.k.a. IBA�s -- as defined by the International Banking Act) report annually, as of December. \| \| --- \| --- \| \|  \| All institutions that have any fiduciary or related assets file this information annually each December. However, Quarterly data is ONLY available for institutions that have total fiduciary assets greater than $250 million AND gross fiduciary and related service income greater than 10% of the revenue from the preceding calendar year. \| |

| **Fiduciary power exercised :** |
| --- |
| Institution or trust company subsidiary of the institution **exercises** fiduciary powers granted by a state or national regulatory authority to administer accounts in a fiduciary capacity. Fiduciary capacity generally means: trustee, executor, administrator, registrar of stocks and bonds, transfer agent, guardian, assignee, receiver, custodian under a uniform gifts to minors act, investment advisor, any capacity in which the institution possesses investment discretion on behalf of another, or any similar capacity.   \| **Note:** \| Domestic offices of foreign banks (a.k.a. IBA�s -- as defined by the International Banking Act) report annually, as of December. \| \| --- \| --- \| \|  \| All institutions that have any fiduciary or related assets file this information annually each December. However, Quarterly data is ONLY available for institutions that have total fiduciary assets greater than $250 million AND gross fiduciary and related service income greater than 10% of the revenue from the preceding calendar year. \| |

**TPIMATOTr : Ratio of Total managed assets in fiduciary accounts to Total assets**

Additional variables of interest for other analyses

| **Total managed assets held in fiduciary accounts: TPIMATOT** |
| --- |
| Market Value of total managed assets held in fiduciary accounts personal trust and agency accounts and investment management agency accounts  Note: Reported as a memoranda item in December only |

| **Total employees (full-time equivalent): numemp** |
| --- |
| The number of full-time employees on the payroll of the bank and its subsidiaries at the end of the quarter. |

| **Total assets : asset** |
| --- |
| The sum of all assets owned by the institution including cash, loans, securities, bank premises and other assets. This total does not include off-balance-sheet accounts. |

| **Total deposits : dep** |
| --- |
| The sum of all deposits including demand deposits, money market deposits, other savings deposits, time deposits and deposits in foreign offices. |

| **Interest-bearing deposits : depi** |
| --- |
| Interest-bearing deposits (includes interest-bearing deposits in foreign offices). |

| **Deposits held in domestic offices: depdom** |
| --- |
| The sum of all domestic office deposits, including demand deposits, money market deposits, other savings deposits and time deposits. |

| **% insured (estimated) : iddepinr** |
| --- |
| Estimated amount of insured deposits as a percent of total deposit liabilities before exclusions (gross) as defined in section 3(l) of the Federal Deposit Insurance Act and FDIC regulations.   Available in the FDIC Institution Directory beginning in March 2009.   NOTE: Although standard FDIC insurance coverage was temporarily raised from $100,000 to $250,000 in October 2008, institutions are required to report the source elements for this estimate based on the $100,000 coverage limit through June 2009. Beginning with the September 30, 2009 reporting period, institutions are required to report based on the $250,000 coverage limit.   RIS definition: IDDEPINR = (DEPINS / DEPBEFEX) *100 |
